# Multiresponsive Isotropic Structural Color in Cholesteric Liquid Crystals

**DOI:** 10.1021/jacs.6c07395

**Published:** 2026-07-25

**Authors:** Mauricio Vera-Arévalo, Alberto Concellón

**Affiliations:** Instituto de Nanociencia y Materiales de Aragón (INMA), Departamento de Química Orgánica, 16765 CSIC-Universidad de Zaragoza, 50009 Zaragoza, Spain

## Abstract

Cholesteric liquid crystals (CLCs) are promising photonic materials due to their tunable Bragg reflection. However, their practical implementation remains constrained by a fundamental trade-off: planar CLC films suffer from intrinsic viewing-angle dependence, whereas CLC emulsions remain restricted in responsiveness and macroscopic processability. Here, we address this limitation by developing multiresponsive CLC emulsion-based materials that generate isotropic (angle-independent) structural color through radial helical organization. A reactive CLC formulation was optimized to incorporate functional motifs responsive to temperature, chemical analytes, and light. The resulting droplets are embedded within a polymer matrix to yield mechanically robust films and printable inks that retain tunable Bragg reflection while suppressing angular color dispersion, enabling clearly discernible macroscopic coloration. These materials can be cast into molds, directly written, or brush-coated onto arbitrary substrates, allowing patterned coatings and three-dimensional architectures with preserved optical response. Across all formulations, distinct and reversible reflection-band shifts are observed under external stimuli, demonstrating that cholesteric pitch modulation is preserved under confinement. This strategy establishes polymer-embedded CLC droplets as versatile, multiresponsive photonic building blocks and offers a generalizable route toward processable, isotropic structurally colored materials capable of translating environmental inputs into accessible optical signals.

## Graphical abstract

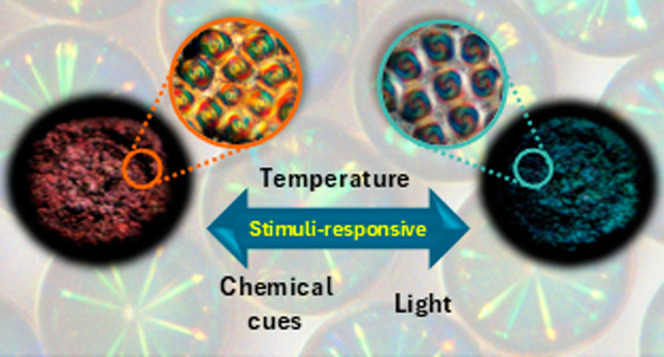

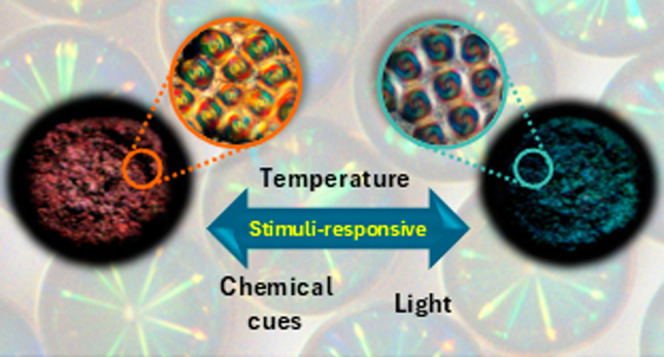

## Introduction

Liquid crystals (LCs) are functional soft materials that combine the long-range anisotropic order of crystalline solids with the fluidity of conventional liquids.^1^ Their ability to respond to diverse external stimuli—including thermal, electrical, photochemical, and mechanical inputs—has enabled widespread applications across display technologies as well as materials science, biology, biomedical engineering, and nanotechnology.^2–5^ This dynamic reconfigurability allows subtle environmental changes to be translated into macroscopic optical responses, establishing LCs as key platforms for adaptive materials.^6–8^ Within the broad family of LC phases, cholesteric liquid crystals (CLCs) occupy a central position owing to the supramolecular chirality present in their molecular organization.^9–12^ When chirality is introduced into a nematic LC host, the director undergoes a periodic rotation, resulting in a helical superstructure that gives rise to selective Bragg reflection.^13,14^ The reflected wavelength (λ) is governed by the helical pitch (*P*), the average refractive index (*n̅*), and the viewing angle (θ), according to Bragg’s law (λ = *P*·*n̅*·cos θ).^15^ As a result, the perceived color is inherently dependent on the observation angle, leading to angular color dispersion in conventional planar cholesteric systems.

The helical pitch (*P*)—and therefore the reflected color—can be tuned by adjusting the concentration of a chiral dopant (*c*), following *P* = 1/*c*·*HTP*, where the helical twisting power (*HTP*) quantifies the efficiency of the dopant in inducing twist within the nematic host.^15^ Beyond dopant concentration, the pitch can be modulated through compositional design or external stimuli, enabling direct optical transduction of environmental changes.^16^ Temperature-responsive CLC formulations capable of tuning their reflection across the visible spectrum have been extensively explored.^11^ More recently, incorporation of functional molecular switches—such as azobenzene derivatives for photoresponsiveness^17–19^ or benzoic acid derivatives for pH and chemical sensing^20–22^ —has significantly broadened their application scope. This high degree of tunability has generated considerable interest in CLCs for optical sensing and responsive photonic technologies, where subtle environmental variations can be translated into measurable chromatic shifts.

However, the angular dependence introduced by θ results in the well-known blue shift observed when the viewing angle deviates from normal incidence, compromising color purity in planar cholesteric systems.^23^ This intrinsic viewing-angle dependence fundamentally limits their implementation in applications requiring isotropic (angle-independent) structural color. To address this challenge, CLCs have been confined within spherical emulsion droplets,^24,25^ where the helical axis preferentially adopts a radial configuration. In such geometries, each droplet behaves as an independent photonic microobject with a largely angle-insensitive optical response.^26–30^ These droplets therefore constitute promising building blocks for smart materials in optics, photonics, sensing, and soft robotics.^31^

Despite these advantages, CLC emulsions are limited by stability issues arising from droplet coalescence, particularly under exposure to air or mechanical stress, which limit the full exploitation of their optical properties. Chemical stabilization using reactive mesogens has emerged as an effective strategy to overcome this issue.^8,32^ Incorporation of polymerizable LC monomers enables the formation of a covalent polymer network upon photoinduced radical polymerization, preserving the cholesteric organization while enhancing mechanical robustness. These polymer-stabilized CLC droplets can be embedded within polymer matrices or processed into freestanding films and architected three-dimensional structures while retaining their structural color.^29,33–43^ However, a critical limitation remains: in contrast to conventional CLC thin films—which have been extensively exploited as one-dimensional photonic materials capable of sensing diverse environmental inputs (e.g., temperature, gases, humidity, solvents, and chemical cues) through measurable shifts in their reflection band—CLC emulsions confined within polymer matrices have largely remained restricted to single-stimulus responses, primarily temperature and mechanical deformation. As a result, comparatively few examples have demonstrated modular cholesteric platforms capable of integrating different chemically responsive functionalities within a common photonic system. This limited stimulus scope constrains their potential for advanced adaptive optical systems capable of detecting a broader spectrum of chemical or environmental signals. Overcoming this limitation therefore remains a key challenge for the development of multifunctional cholesteric photonic architectures.

Herein, we address this limitation by developing angle-independent, multiresponsive photonic platforms based on CLC droplets (Figure 1). A series of photo-cross-linkable CLC formulations incorporating molecular units responsive to temperature, pH, chemical cues, and light were optimized to integrate responsiveness within the helical architecture while preserving its intrinsic photonic properties. Emulsification yields droplets with radial helical organization, producing isotropic structural color. Subsequent photopolymerization locks the cholesteric alignment within each droplet, stabilizing the radial configuration while maintaining tunable Bragg reflection, and the resulting droplets are incorporated into a secondary polymer matrix to afford mechanically robust thin films and printable inks. These inks can be applied by mold casting, direct writing, or brush coating onto arbitrary substrates, enabling patterned coatings and three-dimensional architectures while retaining a largely angle-independent optical response. The CLC emulsion-based platforms exhibit distinct stimulus-induced shifts in their reflection bands—accompanied by perceptible macroscopic color changes—in response to temperature, pH variations, chemical species, and light irradiation. Collectively, this strategy overcomes the limited responsiveness of polymer-dispersed CLC droplets and establishes a generalizable platform for multifunctional, isotropic structurally colored materials in adaptive photonic applications.

**Figure 1:**
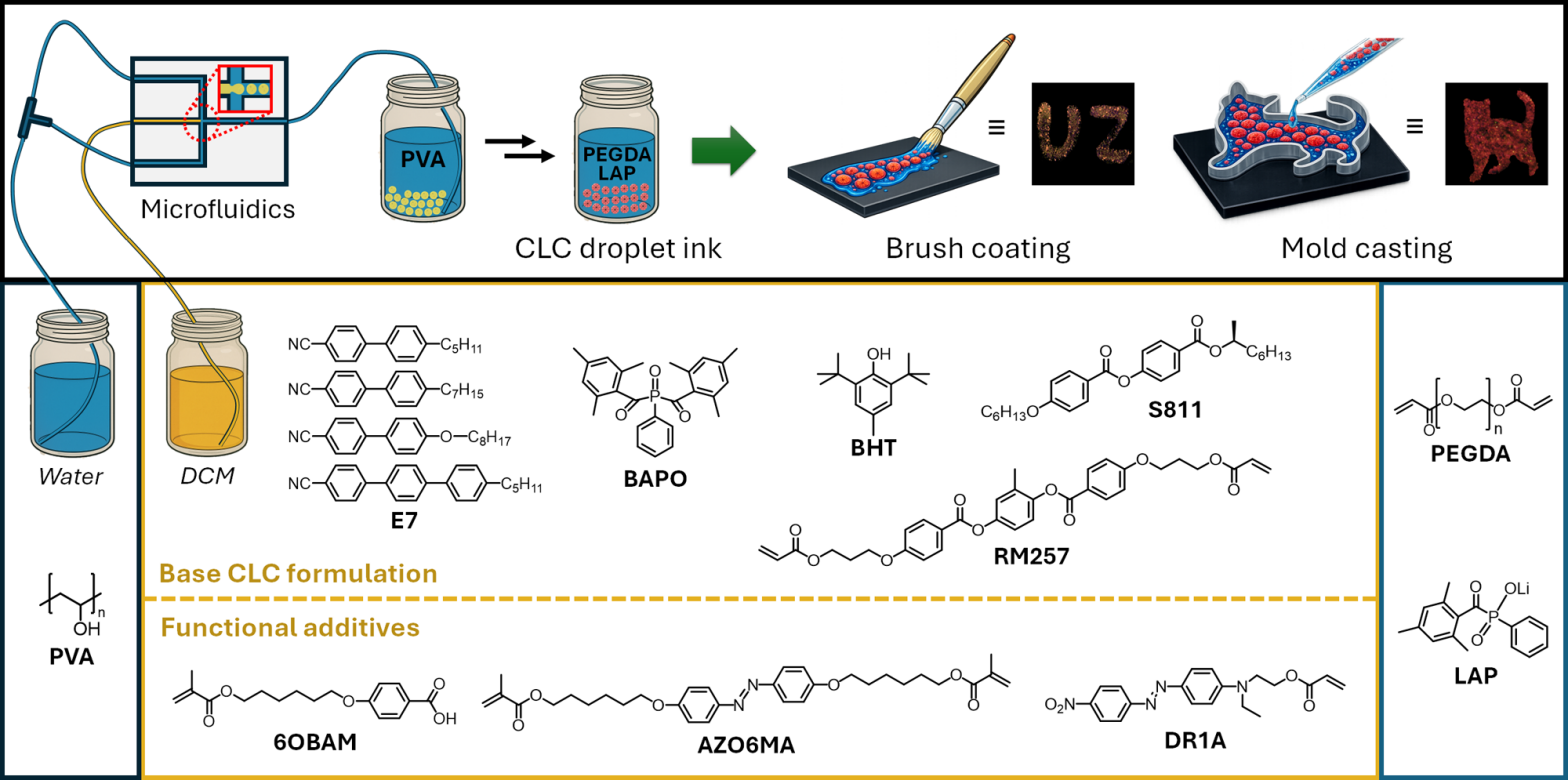
Schematic illustration of the fabrication of isotropic (angle-independent), multiresponsive cholesteric platforms. A photo-cross-linkable CLC formulation is emulsified in an aqueous PVA continuous phase, yielding droplets with radial helical organization. Subsequent photopolymerization stabilizes the cholesteric architecture within each droplet. The resulting polymer-stabilized droplets are incorporated into a PEGDA/LAP continuous phase to form a processable photonic ink, which can be applied by mold casting or brush coating. UV curing produces polymer-embedded CLC architectures with preserved structural color and angle-independent optical response.

## Results and Discussion

### Establishing an Angle-Independent Cholesteric Platform

We first designed a photo-cross-linkable cholesteric formulation based on the nematic mixture **E7**, the chiral dopant **S811**, and the mesogenic diacrylate **RM257**, optimized to yield a red-reflecting cholesteric phase at room temperature while enabling subsequent network formation (Figure 1). The formulation comprised 60 wt % of **E7**, a nonreactive LC that facilitates molecular alignment within the droplets; 20 wt % of the left-handed chiral dopant **S811** (HTP ≈ −11 μm^–1^)^44^ to induce the desired pitch; and 20 wt % of the mesogenic cross-linker **RM257** to generate a stabilizing polymer network upon photopolymerization. The mixture additionally contained 2 wt % of the photoinitiator **BAPO** (absorption range 360–450 nm)^45^ and 0.5 wt % of the thermal stabilizer **BHT** as a radical scavenger. Polarized-light optical microscopy (POM) revealed characteristic cholesteric textures at room temperature (Figure S1), and differential scanning calorimetry (DSC) confirmed a cholesteric phase that transitioned to the isotropic liquid state at 34 °C (Figure S2). After irradiation at 365 nm for 10 min, the mixture forms a polymer-stabilized CLC, in which the polymer network acts as a structural scaffold that stabilizes the cholesteric order without significantly perturbing its intrinsic responsiveness. FTIR spectroscopy showed complete disappearance of the acrylate C=C stretching band at 1640 cm^–1^ and the out-of-plane bending vibration of =C–H at 980 cm^–1^ (Figure S3), efficient polymer network formation upon UV irradiation. DSC analysis of the cross-linked material revealed a slight decrease (ca. 3 °C) in the cholesteric-to-isotropic transition temperature (*T*_*Ch‑I*_) and the appearance of a glass transition (*T*_*g*_) at 17 °C, evidencing formation of the polymer network while largely preserving the cholesteric organization (Figure S2).

To evaluate the intrinsic angular response of this formulation, planarly aligned cholesteric thin films were prepared by dissolving the reactive CLC mixture in dichloromethane (DCM) and depositing it between a methacrylate-functionalized glass substrate and a fluorinated alkylsilane-functionalized glass substrate to promote planar alignment.^46^ After solvent evaporation, the film was photopolymerized under 365 nm UV irradiation to obtain a freestanding CLC polymer coating. Reflection spectra recorded at different viewing angles revealed a well-defined reflection band in the red region under normal incidence (0°) (Figure 2a). As the incident angle increased, the reflection maximum progressively blue-shifted, in agreement with estimated reflection band values calculated using Bragg’s law (Figure S4). This pronounced angular dispersion highlights the intrinsic limitation of planar cholesteric films, in which the reflected color strongly depends on the viewing geometry, and establishes a benchmark for evaluating the angular optical response of CLC emulsion systems.

**Figure 2:**
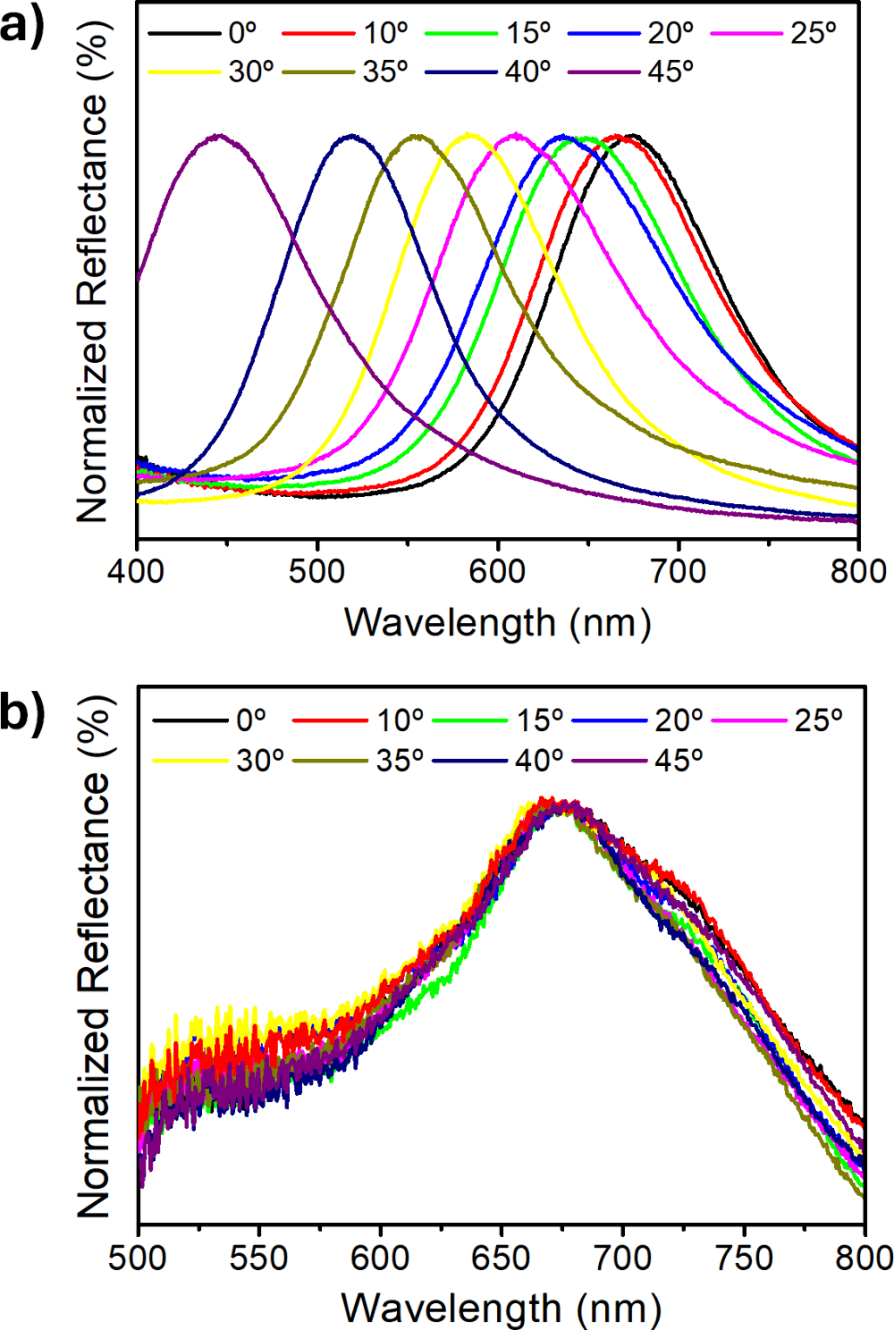
Angle-dependent reflectance measurements of (a) an oriented CLC thin film, and (b) a thin film comprising polymer-embedded CLC droplets. The planar film exhibits pronounced angular dispersion of the reflection band, whereas the droplet-based film largely suppresses angular dependence over the investigated angular range. All spectra are presented as normalized reflectance intensity to facilitate comparison of the reflection wavelength while minimizing variations in absolute reflected intensity arising from unavoidable differences in droplet packing and local film thickness within the CLC droplet-based films.

To overcome the intrinsic viewing-angle dependence of planar cholesteric films, the reactive CLC mixture was dissolved in DCM and emulsified in a 1 wt % aqueous poly­(vinyl alcohol) (PVA) solution. After solvent evaporation, cholesteric droplets were obtained. Bulk emulsification produced polydisperse droplets with diameters of 5–100 μm (Figure S5), whereas microfluidic preparation yielded larger and more uniform droplets with diameters of approximately 200 μm. No significant qualitative differences in response time, spectral shift, or angular optical response were observed across the investigated droplet sizes. However, macroscopic color purity improved significantly with monodisperse droplets, enabling clearer visual observation of the structural color (*vide infra*). POM images revealed a characteristic radial helical configuration of the CLC phase (Figure S6), induced by PVA, which promotes planar anchoring of the LC director at the aqueous interface. This interfacial alignment enforces radial orientation of the helical axis within each droplet. Irradiation at 365 nm for 10 min induced photopolymerization, generating polymer-stabilized CLC droplets in which the cross-linked **RM257** network stabilizes the radial helical structure. Under reflection-mode optical microscopy (Figure 3a), individual droplets exhibited a bright central red spot corresponding to normal Bragg reflection from the radially organized helix, accompanied by weaker radial green reflections arising from the well-known cross-communication phenomenon. This effect originates from off-axis light reflected by one droplet and subsequently redirected toward neighboring droplets, resulting in multiple reflected wavelengths reaching the observer.^34^ This radially organized configuration enables angle-independent reflection, as each droplet behaves as an individual photonic microobject with an orientation-independent optical response, effectively sampling all possible helix orientations with respect to the incident light.

**Figure 3:**
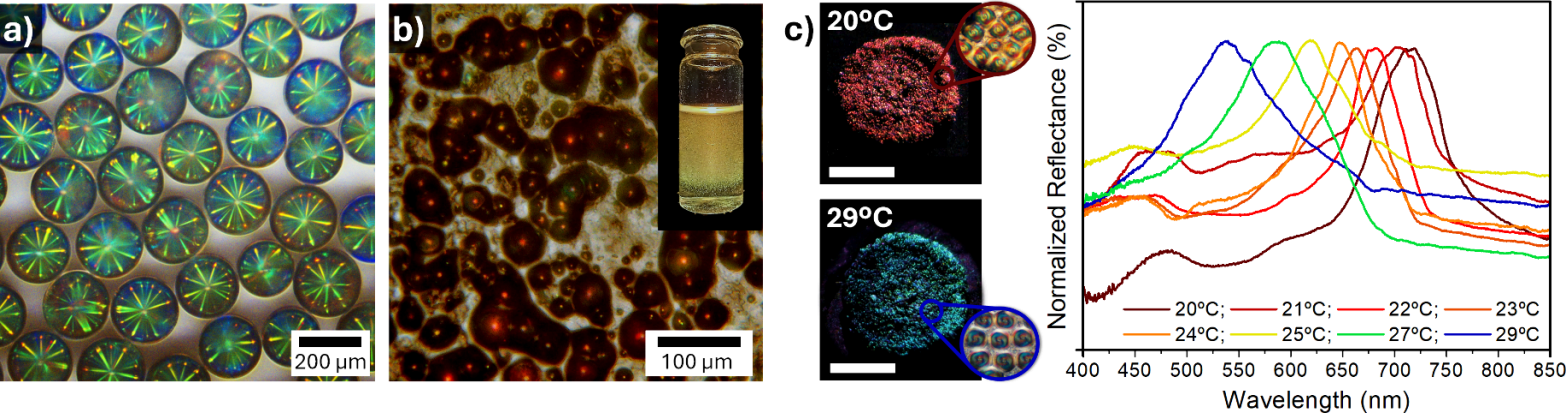
Optical characterization of radially organized CLC droplets and their polymer-embedded films. (a) Reflection-mode optical microscopy image of CLC droplets dispersed in a 1 wt % PVA aqueous solution, exhibiting a characteristic central red reflection arising from radial helical organization. (b) Reflection-mode optical microscopy image of a thin film comprising a monolayer of polymer-embedded CLC droplets, confirming preservation of radial helical alignment and the central red Bragg reflection after embedding. (c) Macroscopic images of polymer-embedded CLC droplet films at different temperatures and corresponding reflectance spectra, showing thermally induced blue shifts of the Bragg reflection while maintaining angle-independent structural color. Scale bar: 0.5 cm.

The polymer-stabilized CLC droplets were subsequently dispersed in a solution containing poly­(ethylene glycol) diacrylate (**PEGDA**, Mn ≈ 6 kDa) and the water-soluble photoinitiator **LAP** (absorption range 300–450 nm), forming a processable emulsion-based ink. **PEGDA** acts as an interdroplet cross-linker, promoting covalent connections between neighboring droplets and thereby enhancing the mechanical integrity of the resulting material^33^ without contributing to the optical response. The formulation was deposited onto a glass substrate confined by a PDMS mold and photopolymerized under 365 nm UV irradiation, yielding thin films consisting of CLC droplets embedded within a cross-linked **PEGDA** matrix. After photopolymerization, the samples were washed with water to remove unreacted aqueous components and subsequently dried to remove the aqueous phase, yielding solid films rather than hydrogel-like materials. Importantly, the radial cholesteric organization of the droplets was fully preserved after embedding (Figure 3b). In striking contrast to the planar films described above, these CLC droplet-based films exhibited only minimal variations in the reflection maximum upon variation of the viewing angle (Figure 2b). This suppression of angular color dispersion arises from spherical confinement of the cholesteric phase, whereby each droplet behaves as an independent radially organized photonic microdomain.^29^ This geometry effectively decouples the macroscopic optical response from sample orientation, enabling largely angle-independent (isotropic) structural color.

We next assessed whether the intrinsic thermoresponsive behavior of the cholesteric phase was preserved. The temperature-dependent evolution of the reflection band was measured for objects comprising polymer-embedded CLC droplets (Figure 3c). Upon heating from room temperature, the pitch decreased due to the negative temperature dependence of the HTP of **S811**,^47^ leading to a progressive blue shift of the reflection band, spanning a significant portion of the visible spectrum within a narrow temperature interval. This thermally induced transition from red to blue was readily observable by the naked eye (Figure 3c), enabling direct visual detection of temperature variations. Despite the thermally induced spectral shifts, the radial helical organization of the droplets remained preserved throughout the investigated temperature range, maintaining the characteristic suppressed angular color dispersion of the system prior to the cholesteric-to-isotropic transition. Above this isotropization temperature, the reflection band disappeared completely, consistent with the loss of cholesteric LC order. In addition to the main reflection band defining the perceived structural color, a broader and less intense secondary band was observed at shorter wavelengths, approximately corresponding to the primary band multiplied by cos.^45^ This secondary band also blue-shifted with increasing temperature and is attributed to the cross-communication phenomenon previously observed in reflection-mode optical microscopy (Figure 3a). Importantly, the optical response was fully reversible and reproducible over multiple heating–cooling cycles (at least 10 cycles) without detectable degradation in optical performance (Figure S7). No significant changes in droplet size or morphology were observed during thermal cycling, consistent with the formation of polymer-stabilized CLC droplets after photo-cross-linking, while the **PEGDA** matrix remained optically and mechanically inert under the applied temperature range. Collectively, these results demonstrate that thermotropic tunability of the cholesteric pitch is fully retained under radial confinement, confirming that polymer stabilization and droplet embedding suppress angular color dispersion without compromising intrinsic thermally driven optical responsiveness.

Beyond thin film geometries, this CLC emulsion-based ink enables fabrication of macroscopically structured 2D and 3D architectures while preserving cholesteric order and structural color. The realization of vividly colored 3D objects from densely packed cholesteric droplets is often hindered by multiple light scattering, which reduces color purity and contrast, particularly in thicker architectures comprising multiple droplet layers. To mitigate incoherent background scattering, a small amount of carbon black (0.05 wt %) was incorporated into the ink, significantly enhancing optical contrast. The modified ink was deposited into three-dimensional molds and photopolymerized in situ under 365 nm irradiation, yielding mechanically stable architectures with preserved structural color. Subsequent washing and drying lead to removal of the aqueous phase, yielding fully solid structures that retain both their shape and optical response. In the presence of carbon black, the resulting architectures exhibited clearly distinguishable red structural color visible to the naked eye, whereas analogous structures prepared without carbon black appeared optically washed out and lacked color contrast due to increased interdroplet scattering (Figure 4a). Reflection spectroscopy of the printed object revealed a well-defined red reflection band (Figure S8), consistent with that observed in thin films, confirming preservation of cholesteric organization during processing. Scanning electron microscopy (SEM) revealed closely packed spherical droplets (Figure 4b), indicating that droplet morphology remains well preserved after processing and cross-linking. The absence of internal contrast within individual droplets further supports compositional homogeneity and effective stabilization of the confined cholesteric phase. Beyond mold-based fabrication, the ink can also be processed via direct writing, injection, or brush painting onto arbitrary substrates, enabling patterned coatings and free-form structures (Figure 4c). These results highlight the versatility of the emulsion-based platform for fabricating structurally colored materials across multiple length scales—from thin films to three-dimensional architectures—while maintaining isotropic optical response.

**Figure 4:**
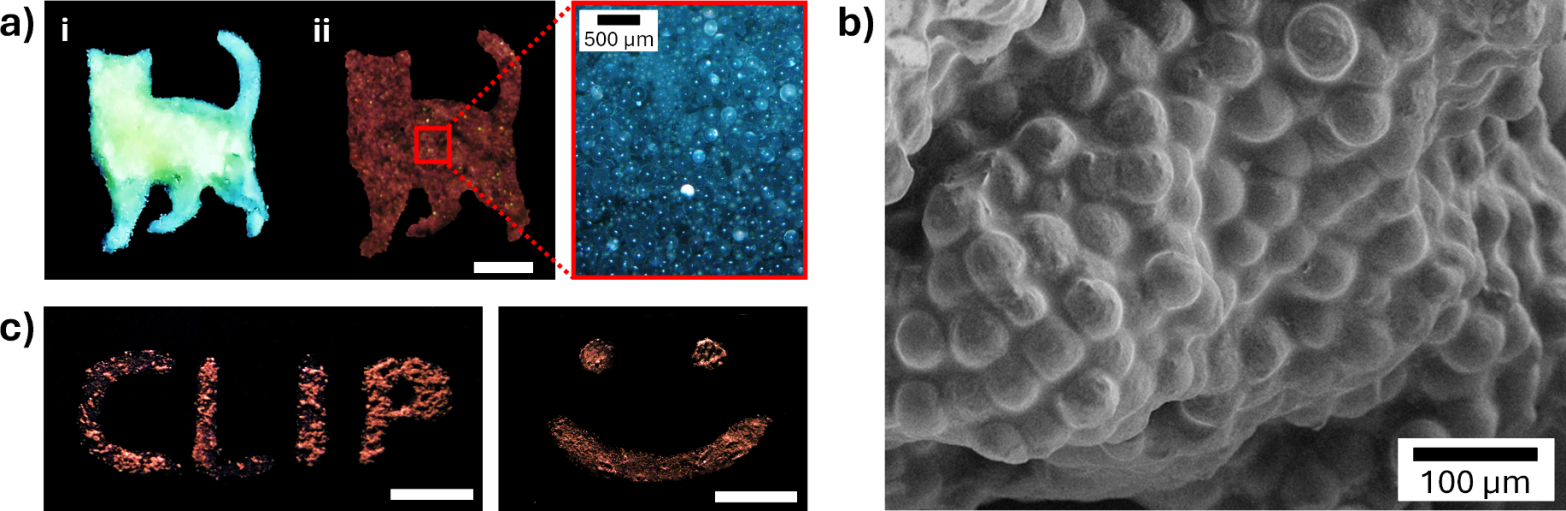
Printable CLC photonic architectures. (a) Cat-shaped 3D objects produced by mold casting of the CLC ink: (i) without carbon black and (ii) with carbon black, yielding vivid red structural color (inset: reflection-mode optical microscopy confirming preserved radial helical organization). Scale bar: 0.5 cm. (b) SEM image showing closely packed polymer-stabilized CLC droplets within the 3D architecture. (c) Free-form patterns generated by brush coating of the photonic ink. Scale bar: 0.5 cm.

### Chemical and Analyte Responsiveness of the Polymer-Embedded Cholesteric Platform

Beyond intrinsic factors that modulate the helical pitch (e.g., temperature or solvent effects), incorporation of functional molecular units enables direct chemical responsiveness within the cholesteric architecture. To impart pH and analyte sensitivity, the benzoic acid derivative **6OBAM**—capable of forming hydrogen-bonded supramolecular cross-links^21,22^ —was incorporated into the starting formulation by replacing 10 wt % of **RM257**. Prior to photopolymerization, POM confirmed the presence of a cholesteric phase at room temperature, while DSC revealed a cholesteric–isotropic transition at 37 °C (Figure S9). After irradiation at 365 nm for 30 min, FTIR spectroscopy showed complete disappearance of the acrylate bands at 980 and 1640 cm^–1^ (Figure S10), confirming successful network formation. The FTIR spectrum additionally revealed an equilibrium between free carboxylic acid groups (≈ 1730 cm^–1^) and hydrogen-bonded carboxylic acid dimers (1700 cm^–1^), the latter acting as reversible supramolecular cross-links.^20^ DSC analysis of the cross-linked material showed a decrease of approximately 8 °C in *T*_*Ch‑I*_ and the appearance of a glass transition at 15 °C (Figure S9), confirming network formation while maintaining a cholesteric phase at room temperature. The modified formulation was successfully emulsified, yielding droplets that preserved the cholesteric organization of the parent platform (Figure S11). Importantly, the resulting droplets retained the angle-independent optical response characteristic of the system, enabling chemical sensing within an isotropic photonic architecture. Films comprising polymer-embedded CLC droplets were then prepared following the procedure described above to evaluate their chemical responsiveness.

To activate the carboxylic acid functionalities, the materials were treated with a 1 M Na_2_HPO_4_/NaH_2_PO_4_ buffer (pH = 8), converting the benzoic acid units into sodium benzoate moieties. FTIR measurements revealed disappearance of the stretching vibration associated with hydrogen-bonded carboxylic acid dimers at 1710 cm^–1^ (Figure S12), while the carbonyl stretching band of the ester groups at 1730 cm^–1^ remained unchanged, indicating that the buffer treatment selectively affected the benzoic acid functionalities without altering the ester moieties. In addition, two new bands appeared at 1544 and 1379 cm^–1^, corresponding to the asymmetric and symmetric stretching modes of COO^–^ groups, consistent with effective deprotonation of the benzoic acid units to form Na^+^-carboxylate species. pH-dependent control experiments further revealed that the onset of the optical response occurred near pH = 4.5–5 (Figure S13), in agreement with the expected p*K*_a_ range of benzoic acid derivatives.

This ionization step induced an initial blue shift of approximately 40 nm, consistent with Na^+^-induced contraction of the cholesteric pitch. To probe binding toward different chemical species, the sodium benzoate-functionalized materials were exposed for 2 h to 0.10 M aqueous solutions of monovalent and divalent cations (K^+^, Ca^2+^, Mg^2+^) as well as the amino acid lysine. Reflection spectra (Figure 5a) revealed analyte-dependent shifts, with K^+^ producing the smallest response and Ca^2+^ inducing the largest blue shift (≈70 nm relative to the initial state), suggesting stronger coordination and greater pitch contraction. FTIR measurements after Ca^2+^ exposure revealed an increase in the symmetric stretching band of COO^–^ from 1379 to 1389 cm^–1^ together with a decrease in the asymmetric stretching band from 1544 to 1532 cm^–1^ (Figure S12), consistent with coordination interactions between carboxylate groups and Ca^2+^ ions. Lysine exhibited an intermediate response, consistent with its charge and molecular dimensions, underscoring the ability of the **6OBAM**-functionalized systems to detect not only metal ions but also other species capable of interacting with carboxylate groups. The limit of detection (LOD) was determined for Ca^2+^ as a representative analyte producing the largest spectral response. The calculated LOD was 0.3 mM (Figure S14). Importantly, the sensing response was fully reversible: treatment with a 1 M NaOAc/AcOH buffer (pH = 4) restored the initial reflection band, and the process could be repeated over five cycles without detectable loss of performance (Figure S15). Although the spectral shifts (≈40–70 nm) were not readily distinguishable by the naked eye (Figure 5a), they were clearly resolved by reflectance spectroscopy, enabling reliable discrimination among different analytes. While the system does not exhibit strict selectivity, the observed analyte-dependent spectral shifts reflect differences in interaction strength with the functional groups. The responsiveness of the droplet-based films is likely to be enhanced relative to planar films due to their high interfacial area, which may promote more efficient partitioning of aqueous analytes, while their isotropic optical response enables more reliable detection independent of viewing angle, facilitating direct spectral readout of these changes.

**Figure 5:**
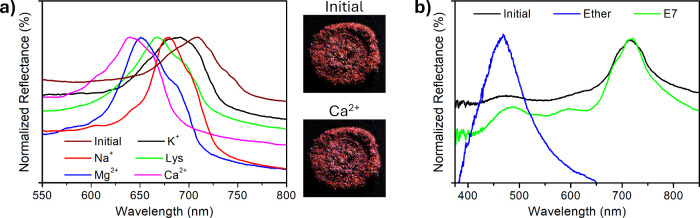
Stimulus-induced spectral modulation and chiral templating in polymer-embedded CLC droplets. (a) Analyte-dependent reflection-band shifts of a **6OBAM**-functionalized CLC droplet film upon exposure to different ionic species and lysine, highlighting coordination-induced contraction of the cholesteric pitch. (b) Reversible solvent-mediated guest exchange: blue shift after ether extraction and recovery of the red reflection band upon reinfiltration with **E7**, demonstrating structural persistence and chiral memory of the cross-linked cholesteric network.

To further probe the structural robustness and templating capability of the cross-linked network, a film comprising polymer-embedded CLC droplets was immersed in diethyl ether. After solvent evaporation at room temperature, the reflection spectrum exhibited a pronounced blue shift (λ ≈ 465 nm), attributed to extraction of the nonreactive nematic mixture **E7** and the chiral dopant **S811** from the droplets. Notably, despite removal of these low-molecular-weight components, the cholesteric order and corresponding structural color were preserved. This behavior indicates that the cross-linked **RM257** network acts as a helical template, maintaining the radial organization of the cholesteric phase even in the absence of the original nonreactive LC constituents. Subsequent reimmersion in **E7** enabled reinfiltration of the nematic molecules into the preorganized polymer scaffold, restoring the reflection band to the red region (Figure 5b).

Circular dichroism (CD) measurements further confirmed retention of supramolecular chirality after solvent extraction (Figure S16), displaying an intense signal with the same handedness as the initial polymer-embedded CLC system. Upon immersion in pure **E7**—without addition of the chiral dopant—not only was the structural color recovered, but the CD signal was also restored. This observation highlights the chiral templating effect of the cross-linked **RM257** matrix, which preserves and transmits helical organization even in the absence of molecular chirality. Collectively, these findings demonstrate that the polymer-stabilized CLC droplets function as structurally persistent cholesteric templates capable of reversible guest exchange and chiral memory without loss of helical organization.

### Light-Induced Modulation of the Polymer-Embedded CLC Droplets

To further expand the versatility of the cholesteric platform toward light responsiveness, two azobenzene-based compounds operating through distinct mechanisms were incorporated into the formulation.

In a first approach, 5 wt % of **RM257** was replaced by the diacrylate azo compound **AZO6MA**, which participates in network formation while introducing photoisomerizable units.^18^ Prior to polymerization, DSC confirmed the presence of a cholesteric phase at room temperature (*T*_*Ch‑I*_ = 46 °C; Figure S17). After photopolymerization under 450 nm irradiation—selected to avoid premature azobenzene isomerization while remaining within the activation window of the **BAPO** photoinitiator—FTIR analysis confirmed complete consumption of the acrylate groups (Figure S18). The cross-linked material exhibited a *T*_*g*_ of 14 °C and a decrease of 22 °C in the *T*_*Ch‑I*_ (Figure S17), indicating a significant modification of network elasticity relative to the parent formulation. Films containing polymer-embedded CLC droplets were prepared as described above. Upon irradiation at 365 nm for 20 min, *trans*–*cis* isomerization of the azobenzene units generated internal stress within the polymer network, leading to elongation of the cholesteric helix and a red shift of approximately 30 nm in the reflection band (Figure 6a). Subsequent irradiation at 450 nm for 20 min induced the reverse *cis*–*trans* isomerization, restoring the initial pitch and reflection wavelength. These results demonstrate that light-induced mechanical modulation of the polymer network is effectively transmitted to the confined cholesteric phase and remains fully reversible over multiple switching cycles. Importantly, the optical response was fully reversible and reproducible over multiple switching cycles (at least 5 cycles) without detectable fatigue or loss of performance (Figure S20).

**Figure 6:**
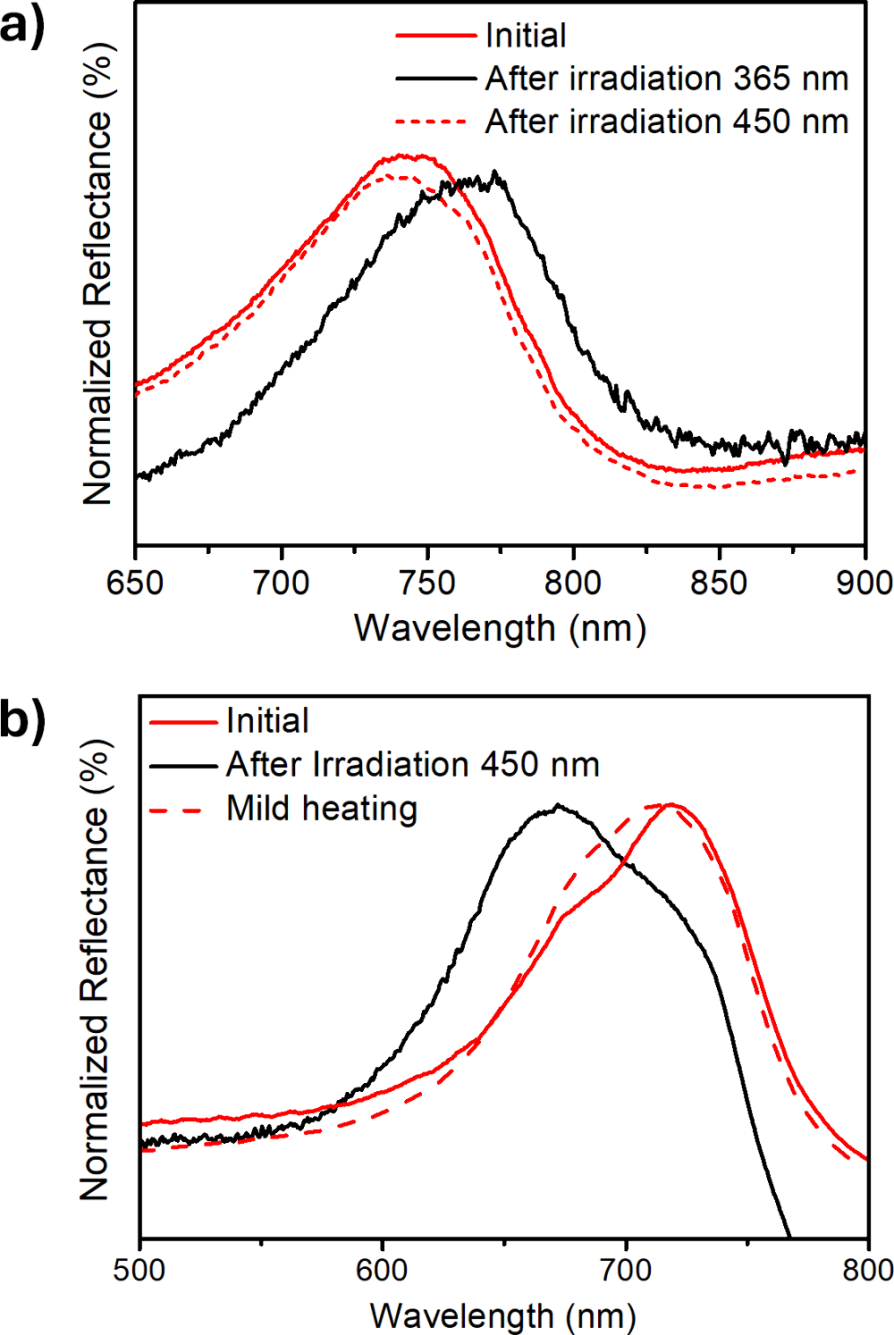
Photoresponsive behavior of polymer-embedded CLC droplets. (a) Reversible red shift of the reflection band in **AZO6MA**-containing films upon 365 nm irradiation and recovery under 450 nm light. (b) Reversible blue shift in **DR1A**-containing films upon 450 nm irradiation, attributed to photothermal heating, followed by recovery upon mild thermal treatment. The 20 min irradiation time was selected because preliminary measurements showed that the reflection-band shift reached a plateau within this period, with no further measurable wavelength changes upon longer irradiation.

As a second strategy, 0.1 wt % of **RM257** was replaced by the monoacrylate azobenzene dye **DR1A**. In contrast to **AZO6MA**, **DR1A** does not significantly contribute to network formation but acts primarily as a photoabsorbing chromophore. **DR1A** exhibits strong absorption between 450 and 600 nm and undergoes *trans*–*cis* isomerization under visible light irradiation, followed by thermally driven relaxation to the *trans* state.^34,48^ Prior to polymerization, DSC confirmed a cholesteric phase at room temperature (*T*_*Ch‑I*_ = 46 °C; Figure S21). After cross-linking (Figure S22), the material displayed a *T*_g_ of 20 °C and a decrease of 12 °C in *T*_*Ch‑I*_ (Figure S21), confirming successful network formation. Films comprising polymer-embedded CLC droplets were fabricated as described above. Upon irradiation at 450 nm for 20 min, *trans*–*cis* isomerization of **DR1A** induced a photothermal effect, resulting in a blue shift of approximately 40 nm in the reflection band (Figure 6b), comparable to the shift observed in the pristine formulation upon a temperature increase of approximately 2 °C. This behavior is consistent with a photothermal mechanism, in which light absorption by **DR1A** is converted into local heating, leading to contraction of the cholesteric pitch. Mild heating to 30 °C followed by cooling restored the initial reflection band, confirming the fully reversible character of the response. The optical response was fully reversible and reproducible over multiple irradiation–relaxation cycles (at least 5 cycles) without detectable fatigue or degradation, demonstrating the robustness of the system (Figure S24). It is noteworthy that the intrinsic orange-red coloration of the azobenzene chromophores partially masks the macroscopic structural color in these formulations. Nevertheless, reflectance spectroscopy clearly resolves the light-induced pitch modulation, confirming that optical responsiveness is retained despite chromophore absorption. Collectively, these results demonstrate that light responsiveness can be integrated into the CLC emulsion-based platform through distinct mechanisms, enabling tunable and reversible isotropic color modulation under light stimuli.

## Conclusions

In conclusion, we establish polymer-embedded cholesteric liquid crystal (CLC) droplets as a robust and versatile platform for angle-independent, stimuli-responsive structural color. By integrating different functional molecular motifs into photo-cross-linkable CLC formulations, we expand the stimulus scope of confined cholesteric systems beyond conventional temperature- or composition-driven pitch modulation, enabling reversible optical responses to pH, chemical species, and light. Emulsification followed by photopolymerization yields droplets with radially organized helices that are permanently stabilized, while embedding within a **PEGDA** matrix affords mechanically robust films and processable inks that preserve Bragg reflection while substantially suppressing angular color dispersion. These materials can be readily processed into patterned coatings and three-dimensional architectures through mold casting or direct deposition techniques, while maintaining their isotropic optical response. Incorporation of a minimal amount of carbon black further enhances optical contrast in bulk structures by suppressing multiple scattering, enabling vivid and well-defined structural coloration at the macroscopic scale. Importantly, across all formulations, the stimulus-induced reflection-band shifts remain fully reversible, demonstrating that radial confinement and polymer stabilization preserve the intrinsic tunability of the cholesteric pitch. In contrast to many previously reported isotropic structural color systems based on fixed photonic architectures, this approach enables postfabrication tunability of the cholesteric pitch within a confined geometry. Overall, these findings establish CLC emulsions as multifunctional photonic building blocks and provide a generalizable strategy for the development of processable, angle-independent colored materials capable of transducing complex environmental inputs into accessible optical signals.

## Supplementary Material



## Notes

Materials and characterization techniques, experimental procedures, supplementary figures (PDF)
