# Supplementary material for: Multiresponsive Isotropic Structural Color in Cholesteric Liquid Crystals
Source: J Am Chem Soc. 2026 Jul 25;148(30):32177–85. doi: 10.1021/jacs.6c07395 (PMC13450381; doi:10.1021/jacs.6c07395)
Supplement: Supplementary file 1 [file ja6c07395_si_001.pdf]

Supporting Information

Multi-Responsive Isotropic Structural Color in Cholesteric Liquid Crystals

Mauricio Vera-Arévalo,[a,b] and Alberto Concellón [a,b,]*

[a]Instituto de Nanociencia y Materiales de Aragón (INMA), CSIC-Universidad de Zaragoza, 50009 Zaragoza, Spain.

[b]Departamento de Química Orgánica, Facultad de Ciencias, Universidad de Zaragoza, 50009 Zaragoza, Spain.

** Author for correspondence: aconcellon@unizar.es*

CONTENTS

1. **Materials and Characterization Techniques**

1.1. Materials

1.2. Instrumentation and Characterization Techniques

2. **Experimental Procedures**

2.1. General Procedure for the Preparation of CLC Emulsions

2.2. Preparation of polymer-embedded CLC droplets

2.3. Preparation of 3D structures

2.4. Preparation of oriented CLC thin films

3. **Supplementary Figures**

1. MATERIALS AND CHARACTERIZATION TECHNIQUES

Materials

Poly(ethylene glycol)diacrylate (PEGDA, M_n 6000) was synthesized following previously reported procedures.[1] Commercial reagents were used as received without further purification: poly(vinyl alcohol) (PVA, M_w 13000-23000, 87–89 % hydrolyzed), sodium phosphate monobasic (NaH_2PO_4), sodium acetate (NaOAc), Dispersed Red 1 (DR1A), phenylbis(2,4,6-trimethylbenzoyl)phosphine oxide (BAPO), 3,5-di-tert-4-butylhydroxytoluene (BHT), lithium phenyl-2,4,6-trimethylbenzoylphosphinate (LAP), 3-(trimethoxysilyl)propyl methacrylate, 1H, 1H, 2H, 2H–perfluorodecyltriethoxysilane, AbsorbMax™ film non-sterile (Sigma-Aldrich); liquid crystal mixture E7, 4,4'-Bis((6-methacryloyloxy)hexyloxy)azobenzene (AZO6MA) (Synthon); 2-Methyl-1,4-phenylene Bis[4-[[6-(acryloyloxy)hexyl]oxy]benzoate] (RM257), S-(+)-2-Octyl 4-(4-hexyloxybenzoyloxy)benzoate (S811) (Cymit); acetic acid glacial (AcOH), dichloromethane (DCM), black carbon acetylene 50% compressed 99.9% (Fisher); L-Lysine monohydrochloride (Alfa Aesar); sodium phosphate dibasic (Na_2HPO_4) (Fluorochem); magnesium chloride 6-hydrate ($MgCl_2\cdot 6H_2O$), potassium chloride (KCl) (Panreac); 4-((6-(Methacryloyloxy)hexyl)oxy)benzoic acid (6OBAM) (BLD pharma).

Instrumentation and Characterization Techniques

Fourier Transform Infrared (FTIR) spectra were recorded using a Bruker Tensor 27 FTIR spectrophotometer. Solid samples were prepared as KBr pellets at approximately 1% by weight. Polarized-light optical microscopy (POM) was conducted using an Olympus BH-2 polarizing microscope with crossed polarizers, equipped with a Linkam THMS600 heating stage connected to a Linkam TMS94 temperature controller. The microscope is fitted with an Olympus DP12 camera controlled by Olympus DP-soft software. Reflection-mode images were acquired using an AmScope ME508 microscope. Thermogravimetric analysis (TGA) was performed on a TA Instruments TGA Q5000IR thermobalance, operating at a rate of 10ºC/min under a nitrogen atmosphere. Differential scanning calorimetry (DSC) was carried out on a TA Instruments Q20 equipment using powdered samples (2-5 mg) sealed in aluminum capsules. Glass transition temperatures (T_g) were determined at the inflection point, and first order transition temperatures were determined at the onsets of the peaks. Circular dichroism (CD) was performed using a Jasco J-810 spectropolarimeter. CD spectra recorded at different rotation angles around the light beam showed identical traces and were subsequently averaged to eliminate linear dichroism artefacts. Reflectance spectra were acquired with an Avantes AvaSpec-ULS2048CL-EVO spectrometer coupled to an AvaLight-HAL-S-Mini non-polarized light source via a fiber-optic cable controlled by AvaSoft 8 software. Monodisperse droplets were produced via Dolomite Microfluidics' Mitos P-Pumps connected to a Bambi PT5 Compressor controlled by Dolomite Flow Control Center software. Scanning electron microscopy (SEM) was performed using a field-emission scanning electron microscope (Carl Zeiss MERLIN Microscopy) at a primary electron energy of 5 kV. Prior to imaging, the 3D printed structures were sputter coated with a 12 nm layer of Pt/Pd (80:20). Images were acquired using a Canon EOS M50 Mark II camera equipped with a Canon EF 50 mm F1.8 STM lens under illumination from an AvaLight-HAL-S-Mini non-polarized light source. The limit of

---

[1] K. H. Son, J. W. Lee, "Synthesis and Characterization of Poly(Ethylene Glycol) Based Thermo-Responsive Hydrogels for Cell Sheet Engineering" *Materials* **2016**, *9*, 854.

detection (*LOD*) was calculated based on the standard deviation of the response of the curve (σ; i.e., the standard deviation of y-intercepts of regression lines) and the slope of the calibration curve (*S*), according to the formula: $LOD = 3.3 \cdot \sigma/S$. σ and S values were obtained from a regression analysis of the calibration curve in Microsoft Excel.

2. EXPERIMENTAL PROCEDURES

General Procedure for the Preparation of CLC Emulsions

The starting-point CLC mixture was prepared by combining 60 wt. % of the LC mixture **E7**, 20 wt. % of the reactive mesogenic monomer **RM257**, and 20 wt. % of the chiral dopant **S811**. The formulation additionally contained 2 wt. % of the photoinitiator **BAPO** and 0.5 wt. % of the radial scavenger **BHT**. Advanced stimuli-responsive mixtures were subsequently prepared by substituting a portion of **RM257** with the compounds **6OBAM**, **AZO6MA**, or **DR1A**, as detailed in **Table S1**.

Table S1. Advanced stimuli-responsive formulations.

Formulation	RM257 (%)	Stimuli-responsive compound (%)
With **6OBAM**	10	10
With **AZO6MA**	15	5
With **DR1A**	19.9	0.1

The dispersed phase was obtained by dissolving the corresponding LC mixture in dichloromethane (DCM) at a concentration of 200 mg/mL. The aqueous continuous phase consisted of deionized water (resistivity of 18.2 MΩ·cm) containing 1 wt. % **PVA** surfactant. First, emulsions were fabricated through bulk emulsification, generating polydisperse droplets with diameters ranging from 5 to 100 μm. In typical preparation, 30 μL of the dispersed phase was emulsified into 500 μL of the aqueous continuous phase using a vortex mixer at 3000 rpm for 5 seconds. The DCM was then gradually evaporated at room temperature by orbital shaker. Secondly, maintaining the same concentrations of the dispersed and continuous phases, monodisperse droplets were generated via microfluids (dispersed phase at 200 mbar and continuous phase at 500 mbar) with sizes typically of 200 μm.

Preparation of polymer-embedded CLC droplets

The CLC droplets obtained, either by bulk emulsification or microfluids, were incorporated into a solution containing 7.5 wt. % **PEGDA** crosslinker and 0.25 wt. % **LAP** photoinitiator. A small amount of black carbon (0.05 wt. %) was added to improve color quality. The resulting emulsion-based ink was deposited onto glass limited by a polydimethylsiloxane (PDMS) template and photopolymerized under 365 nm UV lamp, yielding a thin film comprising a polymer-embedded CLC droplets.

Preparation of 3D structures

Different polyethylene terephthalate (PET) templates were filled with the emulsion-based ink and photopolymerized under 365 nm UV lamp, yielding 3D objects composed of polymer-embedded CLC droplets.

Preparation of oriented CLC thin films

Methacrylate and fluorinated alkylsilane coated functionalized glass substrates were prepared adapting the procedure reported by J. E. Stumpel *et al*.[2] Glass substrates were first cleaned by sonicating in ethanol for 30 min and subsequently treated with Piezobrush PZ3 for 5 s to activate their surfaces.

The activated substrates were then modified by spin coating (3000 rpm, 45 s) either with a 3-(trimethoxysilyl)propyl methacrylate solution (1 vol. % in a 1:1 water-isopropanol mixture) or with a 1H, 1H, 2H, 2H–perfluorodecyltriethoxysilane solution (1 vol. % in ethanol). The coated substrates were cured at 100ºC for 10 min to obtain methacrylate functionalized and fluorinated alkylsilane functionalized glass substrates, respectively.

1 g of the CLC mixture was dissolved in 4 mL of DCM. Approximately 20 µL of this solution was deposited onto a methacrylate functionalized 2.5 x 2.5 cm glass slide. After evaporating the solvent by heating at 70ºC, the slide was cooled to room temperature, and a 2.5 x 2.5 cm fluorinated alkylsilane functionalized glass substrate was placed on top and sheared in one direction to obtain a planarly aligned red film. The film was then photopolymerized under 365 nm UV light (exposure time depends on the formulation and is specified in the Results and discussion section). After curing, the upper glass substrate was removed to yield the orientated CLC thin film.

Reflectance measurements

All measurements were carried out under carefully controlled conditions while maintaining a constant sample-to-detector distance of 2 cm. Reflectance spectra were normalized according to $I_{norm}(\lambda) = I(\lambda)/I_{max}$. Consequently, the maximum normalized reflectance intensity of each spectrum is equal to 1. This normalization was applied to minimize intensity variations arising from unavoidable differences between samples. Unlike CLCs confined in cells, where the sample thickness can be accurately controlled, the polymer-embedded CLC films consist of densely packed CLC droplets rather than continuous planar layers. As a result, local variations in film thickness, together with differences in droplet packing and droplet-substrate contact geometry, influence the absolute reflected intensity without altering the intrinsic spectral response of the CLC droplets. Therefore, normalization was employed to facilitate direct comparison of the spectral shape and the position of the reflection band while minimizing the influence of these experimental variations.

[2] J. E. Stumpel, E. R. Gil, A. B. Spoelstra, C. W. M. Bastiaansen, D. J. Broer, A. P. H. J. Schenning, "Stimuli-Responsive Materials Based on Interpenetrating Polymer Liquid Crystal Hydrogels" *Adv. Funct. Mater.* **2015**, *25*, 3314–3320.

3. SUPPLEMENTARY FIGURES

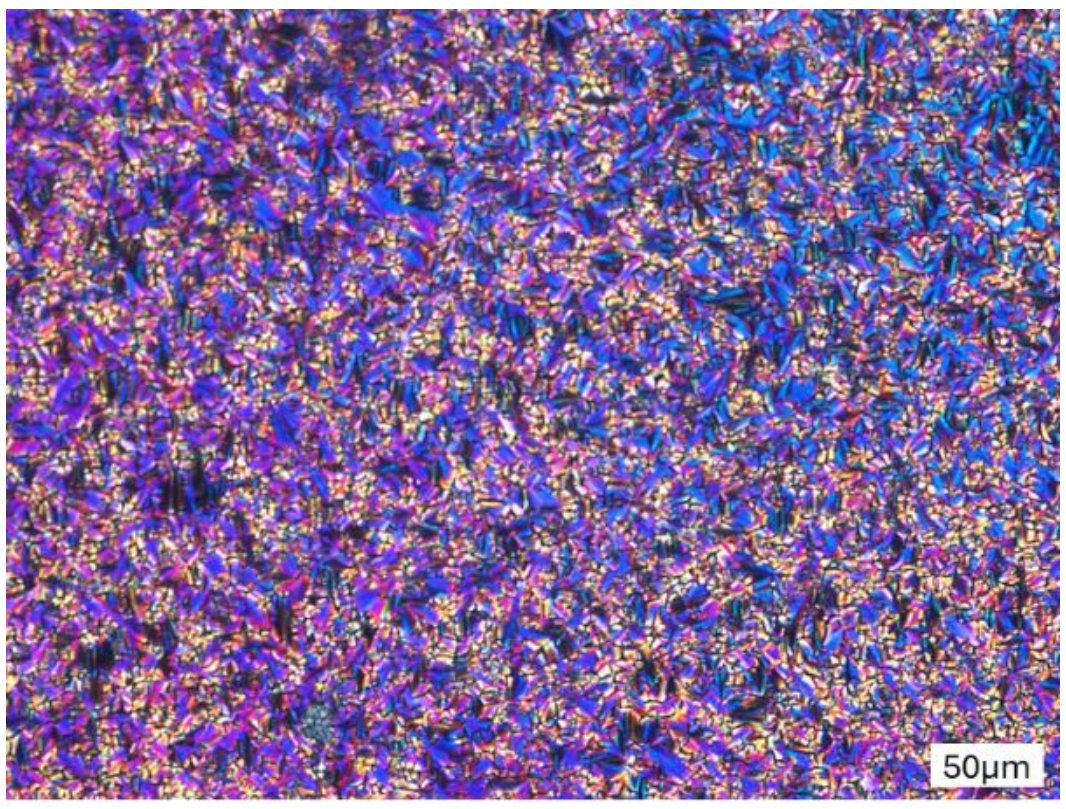

Figure S1. Micrograph of the texture observed by POM under crossed polarizers (25ºC). LC mixture composed of E7 (60 wt. %), RM257 (20 wt. %), S811 (20 wt. %), BAPO, and BHT.

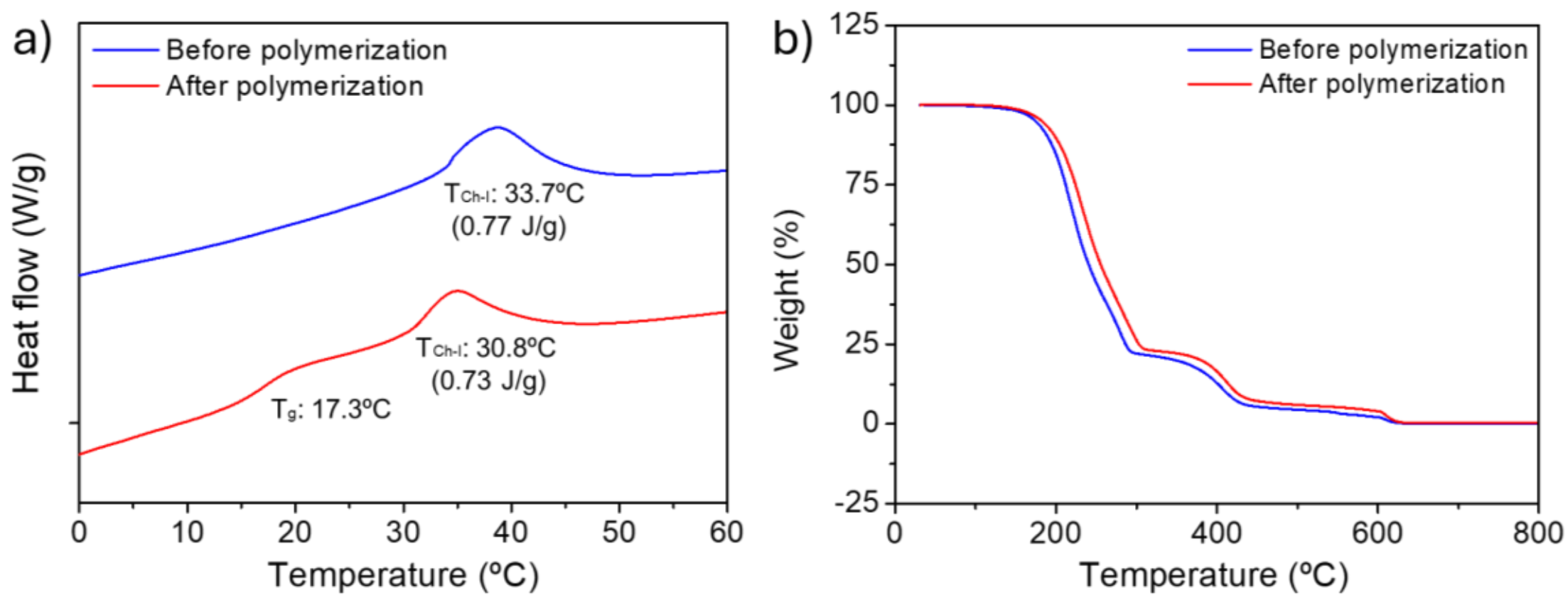

Figure S2. a) DSC traces (10ºC/min, exo down) of a LC mixture composed of E7 (60 wt. %), RM257 (20 wt. %), S811 (20 wt. %), BAPO, and BHT before (blue) and after (red) photopolymerization. **b)** TGA traces of the mixture in a) before (blue) and after (red) photopolymerization.

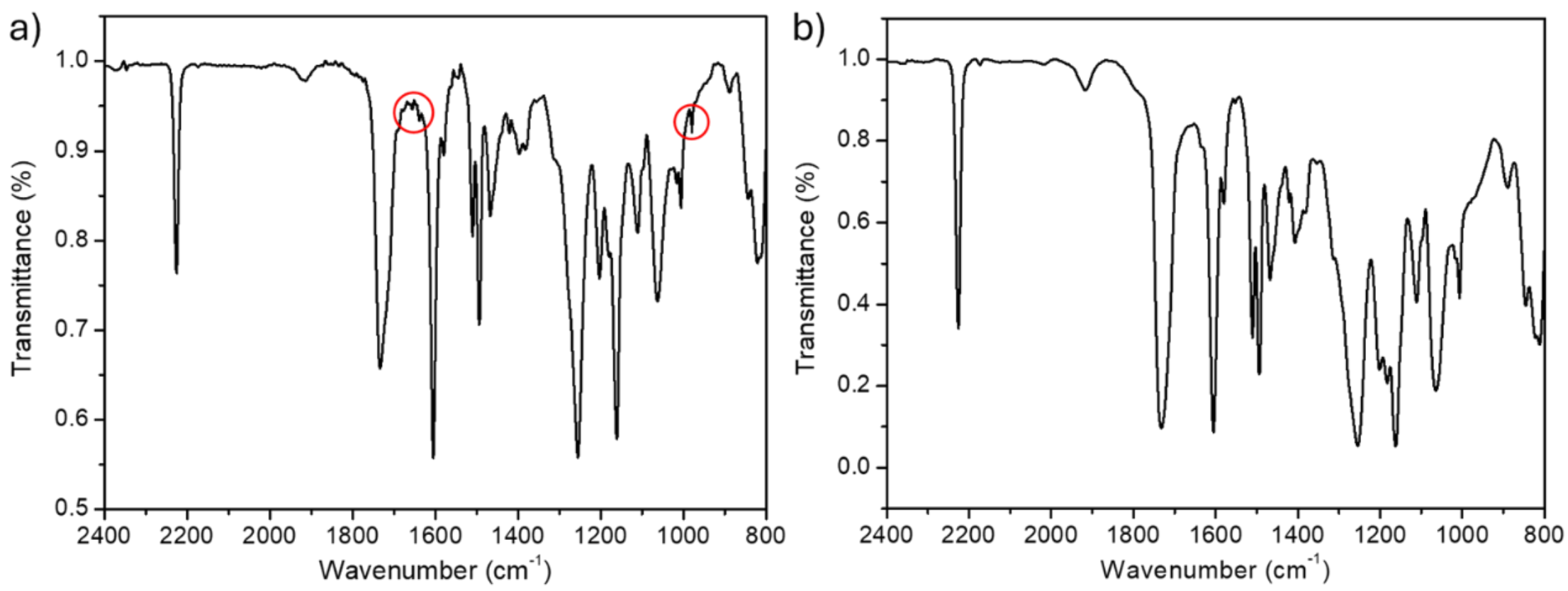

Figure S3. FTIR spectra of: **a)** a LC mixture composed of E7 (60 wt. %), RM257 (20 wt. %), S811 (20 wt. %), BAPO, and BHT before photopolymerization. **b)** the same mixture as in a) after photopolymerization.

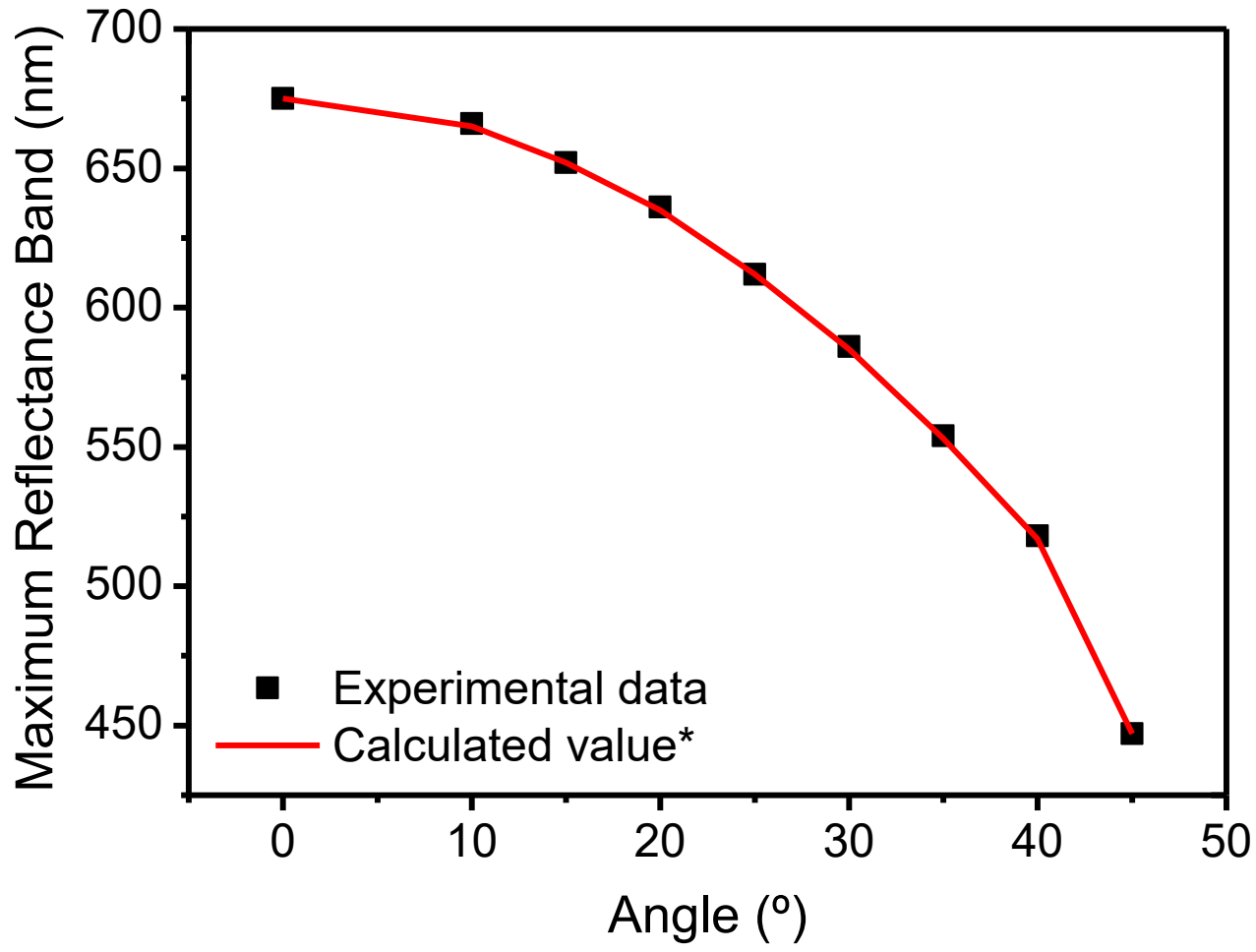

Figure S4. Angle-dependent measurements of the maximum reflectance band as function of the viewing angle of an oriented CLC thin film. *Calculated value for the reflection band was estimated by Bragg's reflection law ($\lambda_\theta = \lambda_{max} \cdot \cos(\theta)$. Mixture composition: E7 (60 wt. %), RM257 (20 wt. %), S811 (20 wt. %), BAPO, and BHT.

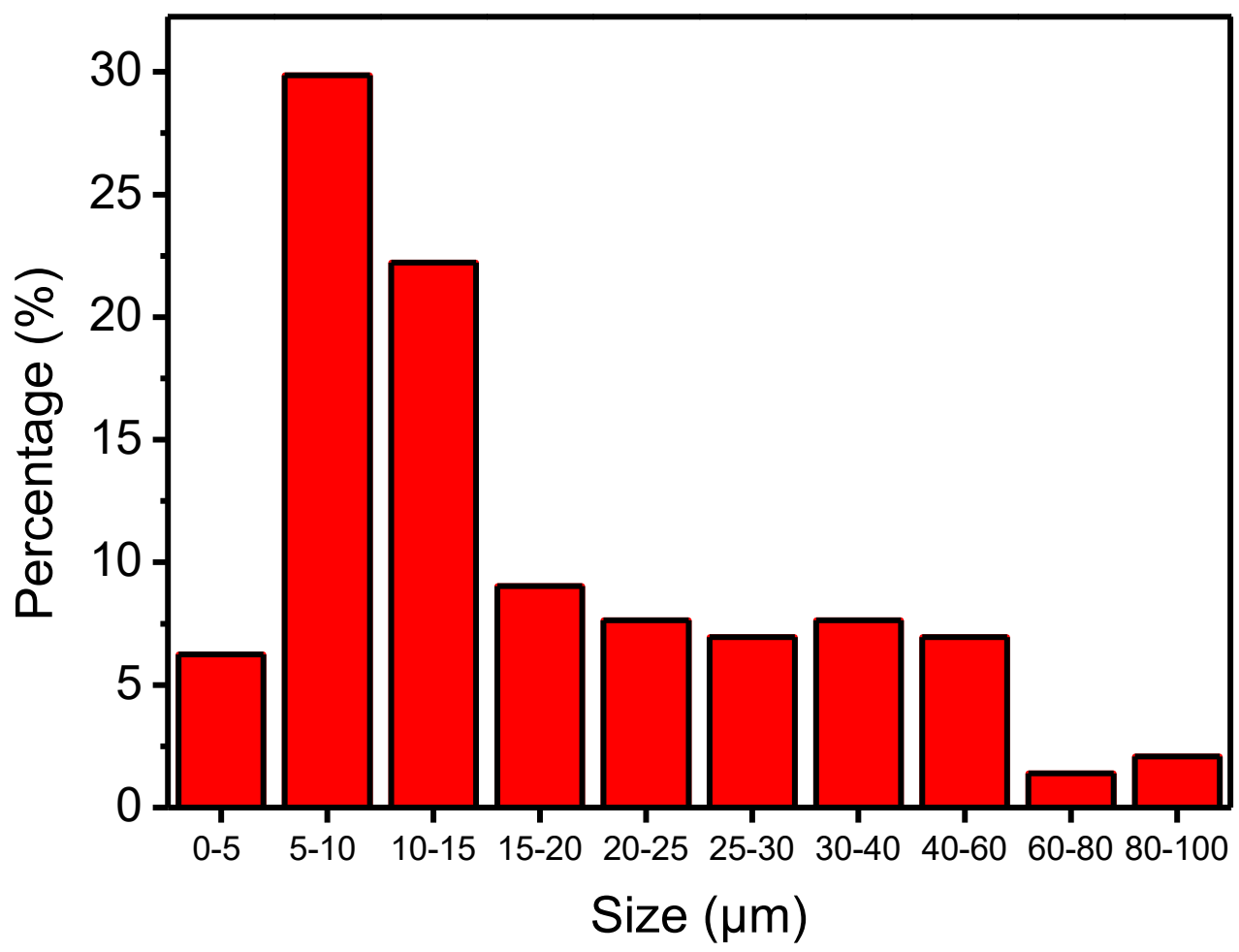

Figure S5. Droplet size distribution of droplets produced by bulk emulsification. Mixture composition: E7 (60 wt. %), RM257 (20 wt. %), S811 (20 wt. %), BAPO, and BHT.

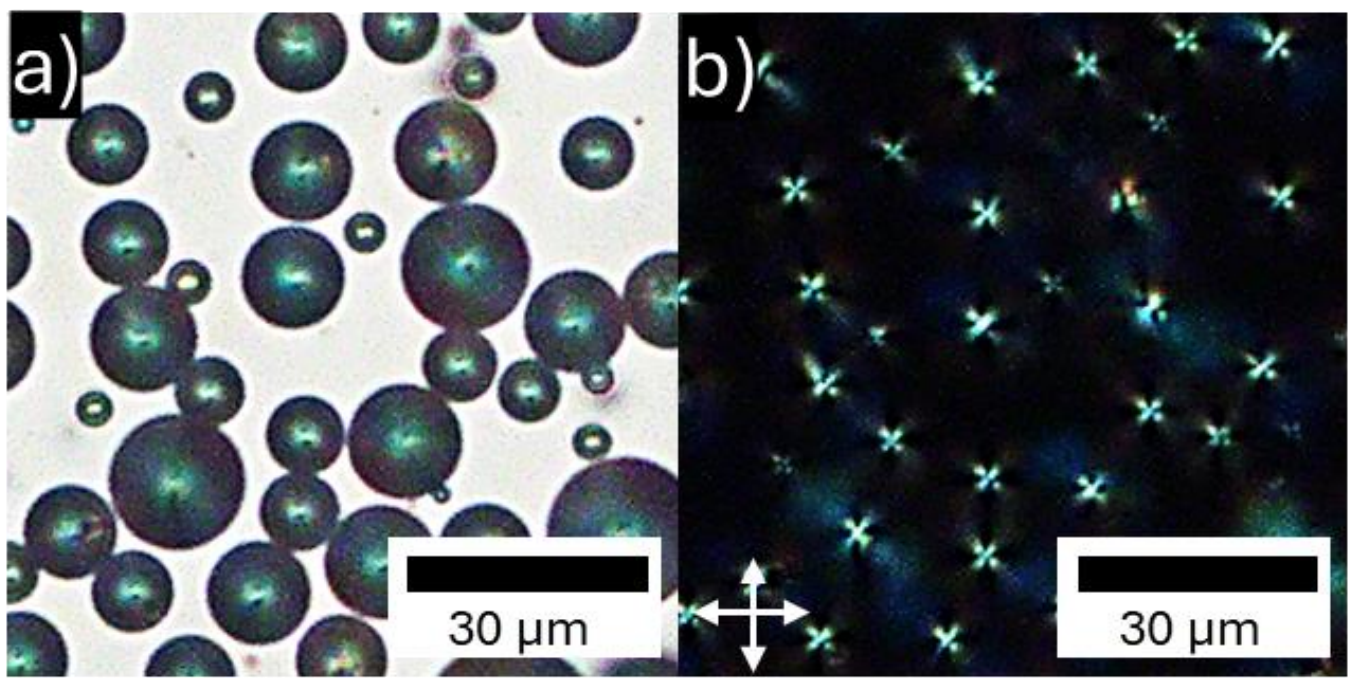

Figure S6. **a)** POM image of CLC emulsions prepared from a mixture of E7 (60 wt. %), RM257 (20 wt. %), S811 (20 wt. %), BAPO, and BHT dispersed in a 1 wt. % PVA aqueous solution. **b)** same sample as in a) observed between crossed polarizers.

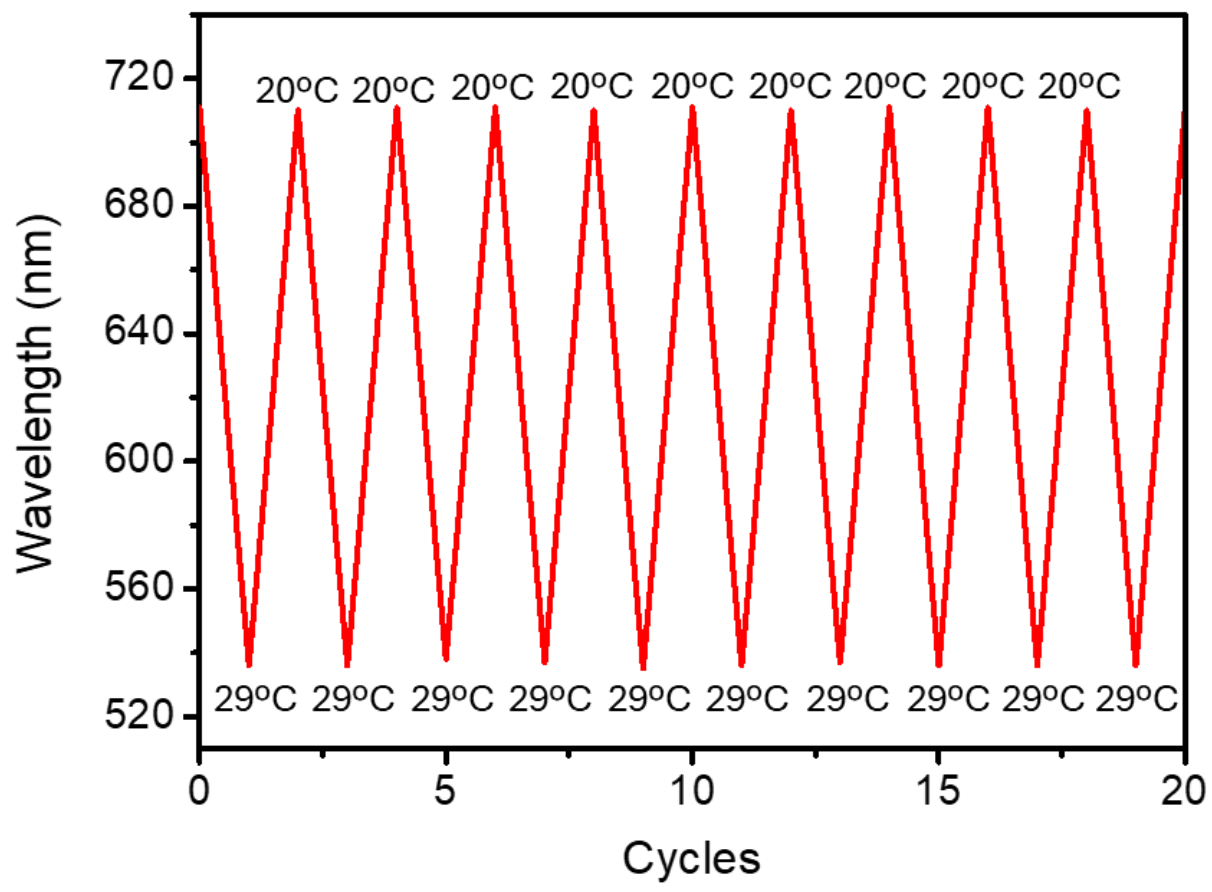

Figure S7. Maximum reflection band evolution during repeated temperature cycling between 20 and 29°C. LC mixture composed of E7 (60 wt. %), RM257 (20 wt. %), S811 (20 wt. %), BAPO, and BHT.

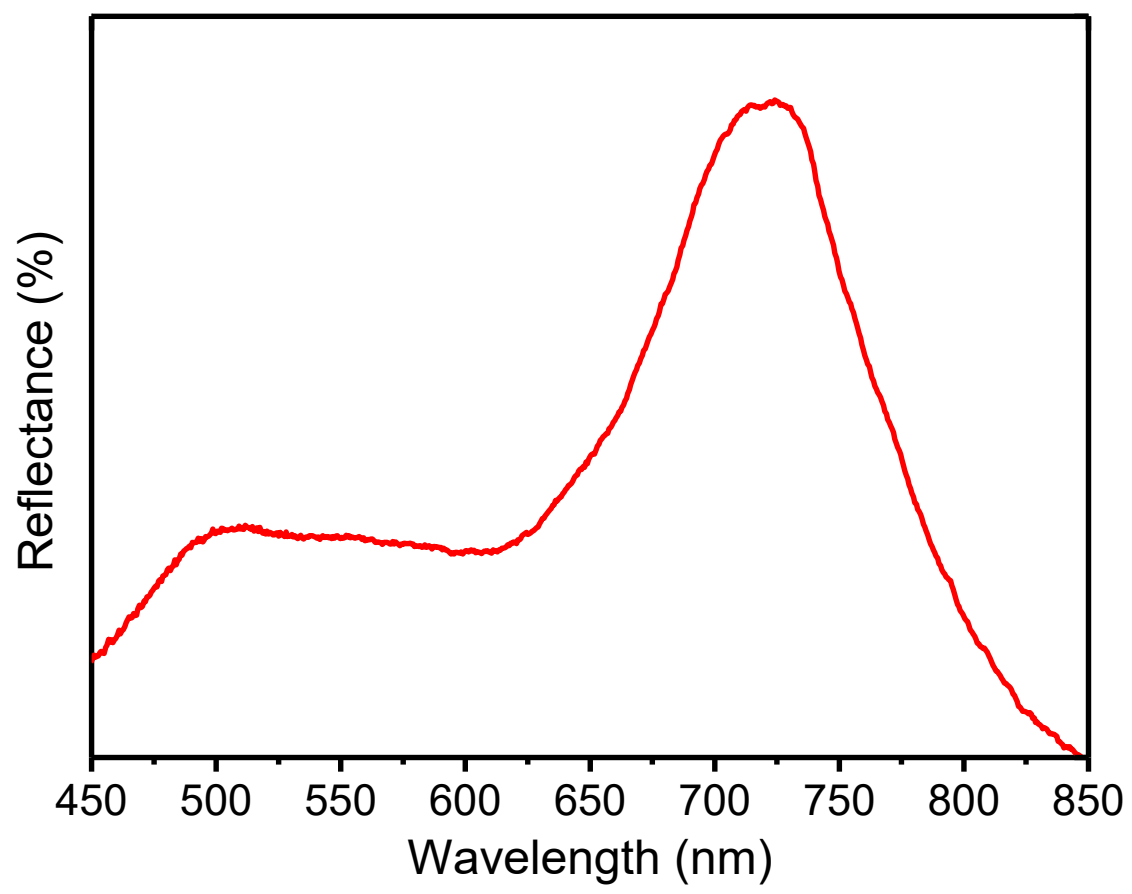

Figure S8. Reflectance spectrum of the Cat-shaped 3D object produced by mold casting. The band around 500 nm corresponds to cross-communication, whereas the band at 700 nm corresponds to Bragg reflection.

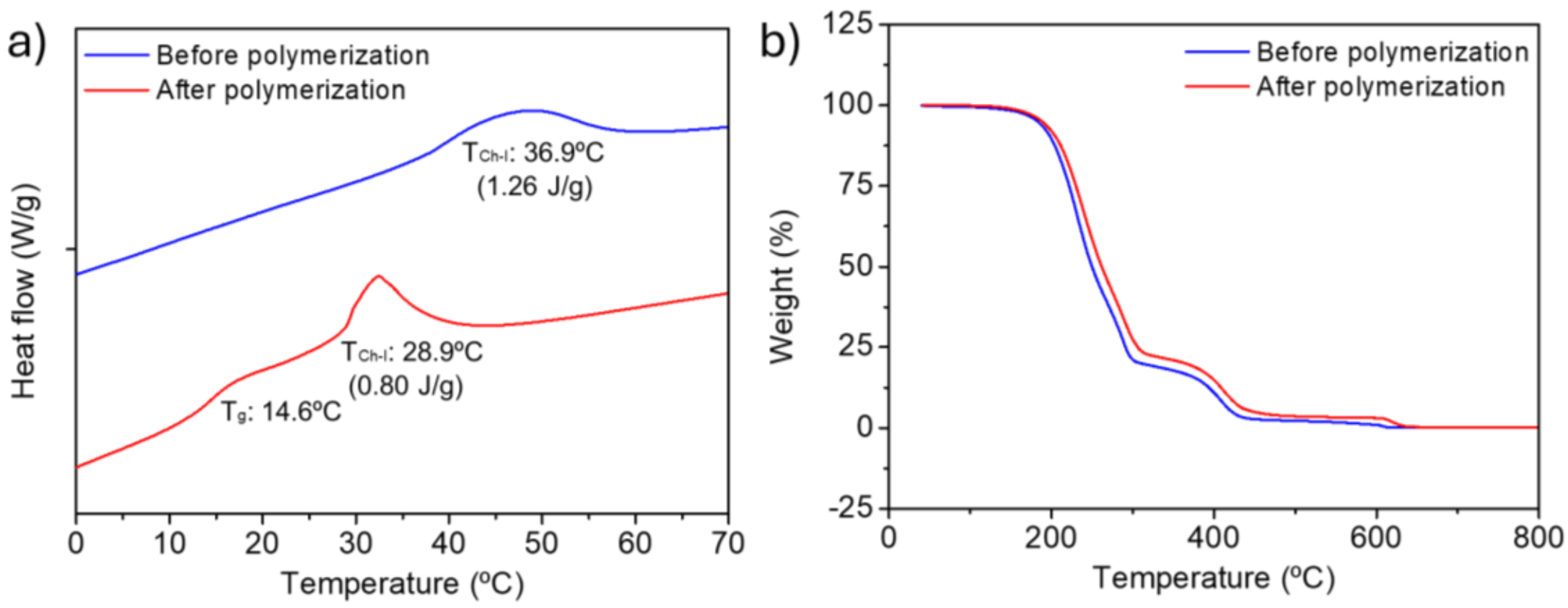

Figure S9. **a)** DSC traces (10°C/min, exo down) of a LC mixture of E7 (60 wt. %), S811 (20 wt. %), RM257 (10 wt. %), 6OBAM (10 wt. %), BAPO, and BHT before (blue) and after (red) photopolymerization. **b)** TGA traces of the mixture in a) before (blue) and after (red) photopolymerization.

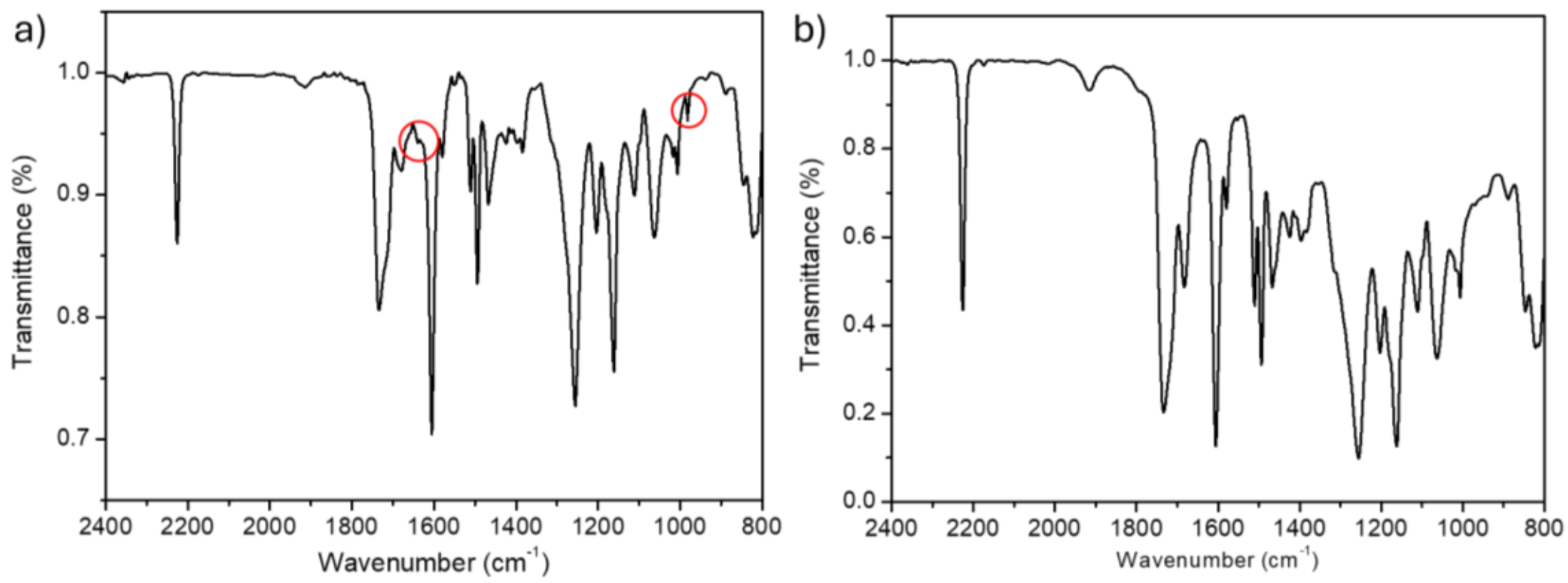

Figure S10. FTIR spectra of: **a)** a LC mixture of E7 (60 wt. %), S811 (20 wt. %), RM257 (10 wt. %), 6OBAM (10 wt. %), BAPO, and BHT before photopolymerization. **b)** the same mixture as in a) after photopolymerization.

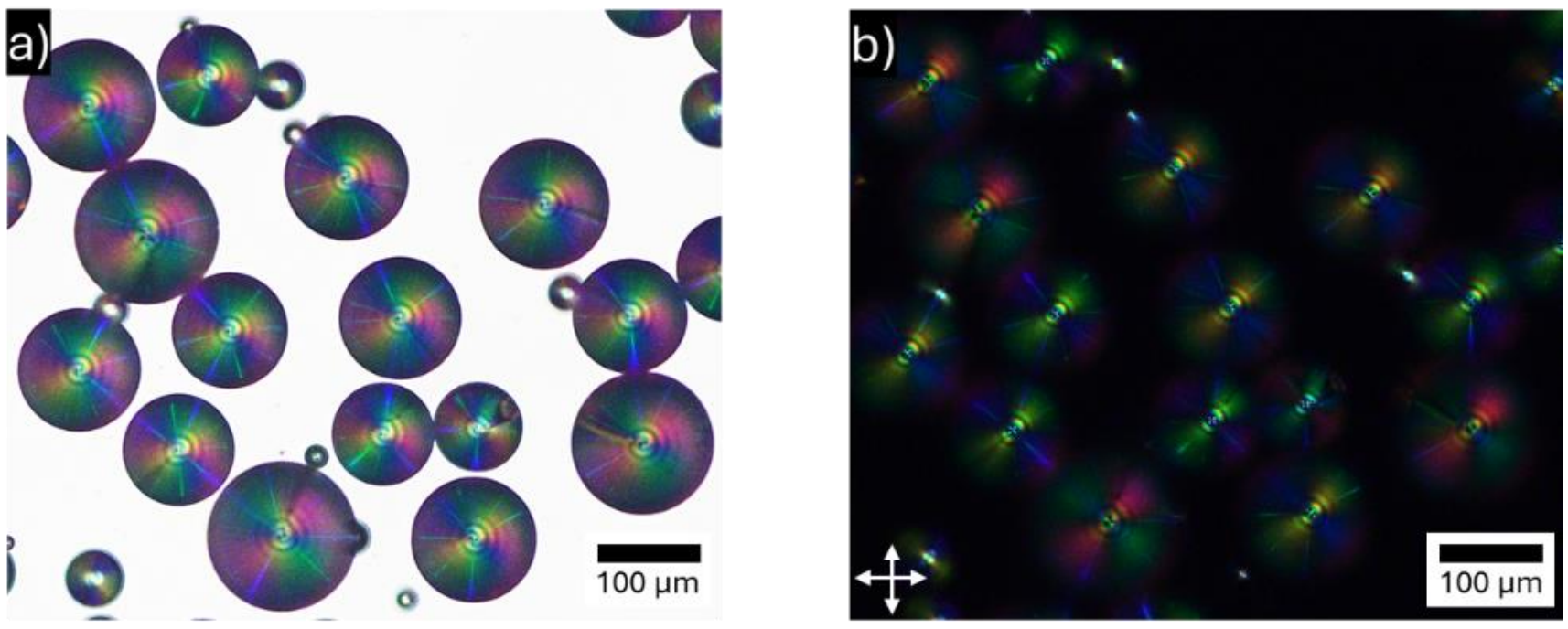

Figure S11. POM images of the pH and analyte responsive CLC emulsions in a 1 wt. % PVA aqueous solution with a) and without b) crossed polarizers. Mixture: E7 (60 wt. %), S811 (20 wt. %), RM257 (10 wt. %), 6OBAM (10 wt. %), BAPO, and BHT.

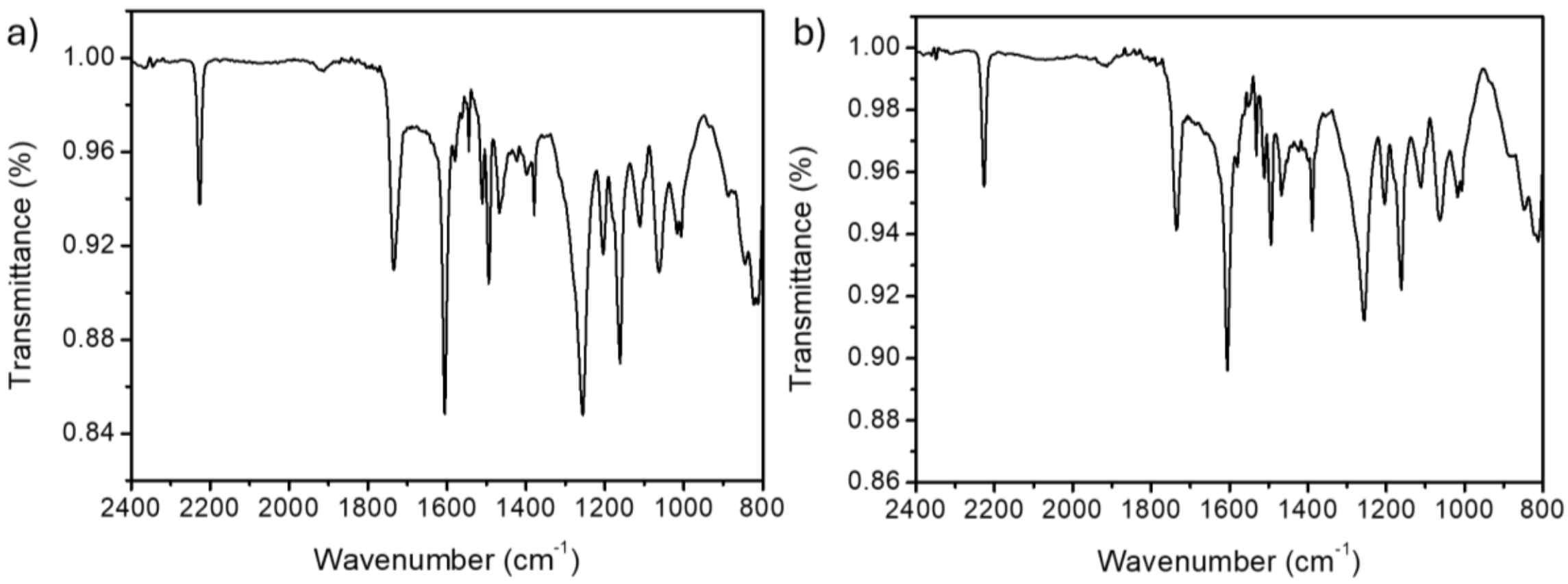

Figure S12. FTIR spectra of **a)** LC mixture shown in **Figure S10** after immersion in 1 M Na_2HPO_4/NaH_2PO_4 buffer solution (pH=8), and **b)** the same mixture as in a) after immersion in 0.10 M Ca^{2+} aqueous solution.

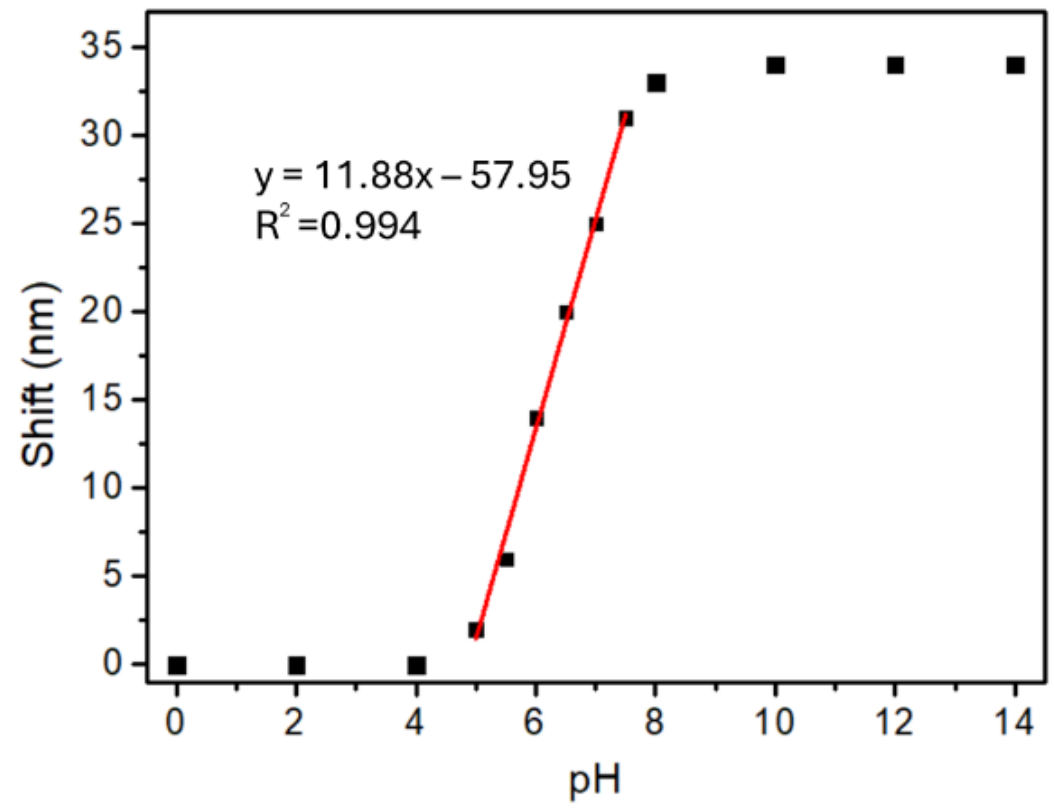

Figure S13. pH calibration curve: shift as function of the pH keeping constant the ionic strength using NaCl. Mixture: E7 (60 wt. %), S811 (20 wt. %), RM257 (10 wt. %), 6OBAM (10 wt. %), BAPO, and BHT.

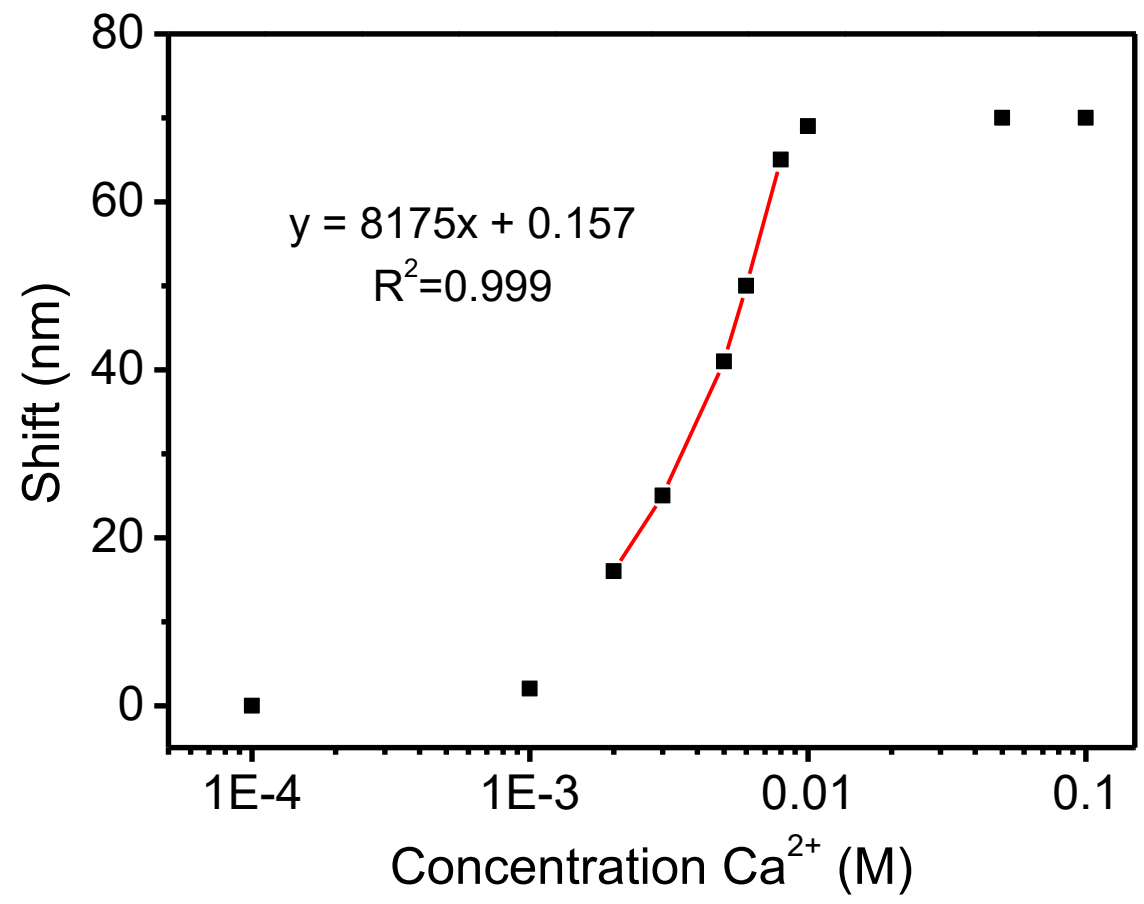

Figure S14. Calibration curve for the calculation of the Limit of Detection (LOD) and Limit of Quantification (LOQ) for Ca^{2+} solutions. Mixture: E7 (60 wt. %), S811 (20 wt. %), RM257 (10 wt. %), 6OBAM (10 wt. %), BAPO, and BHT.

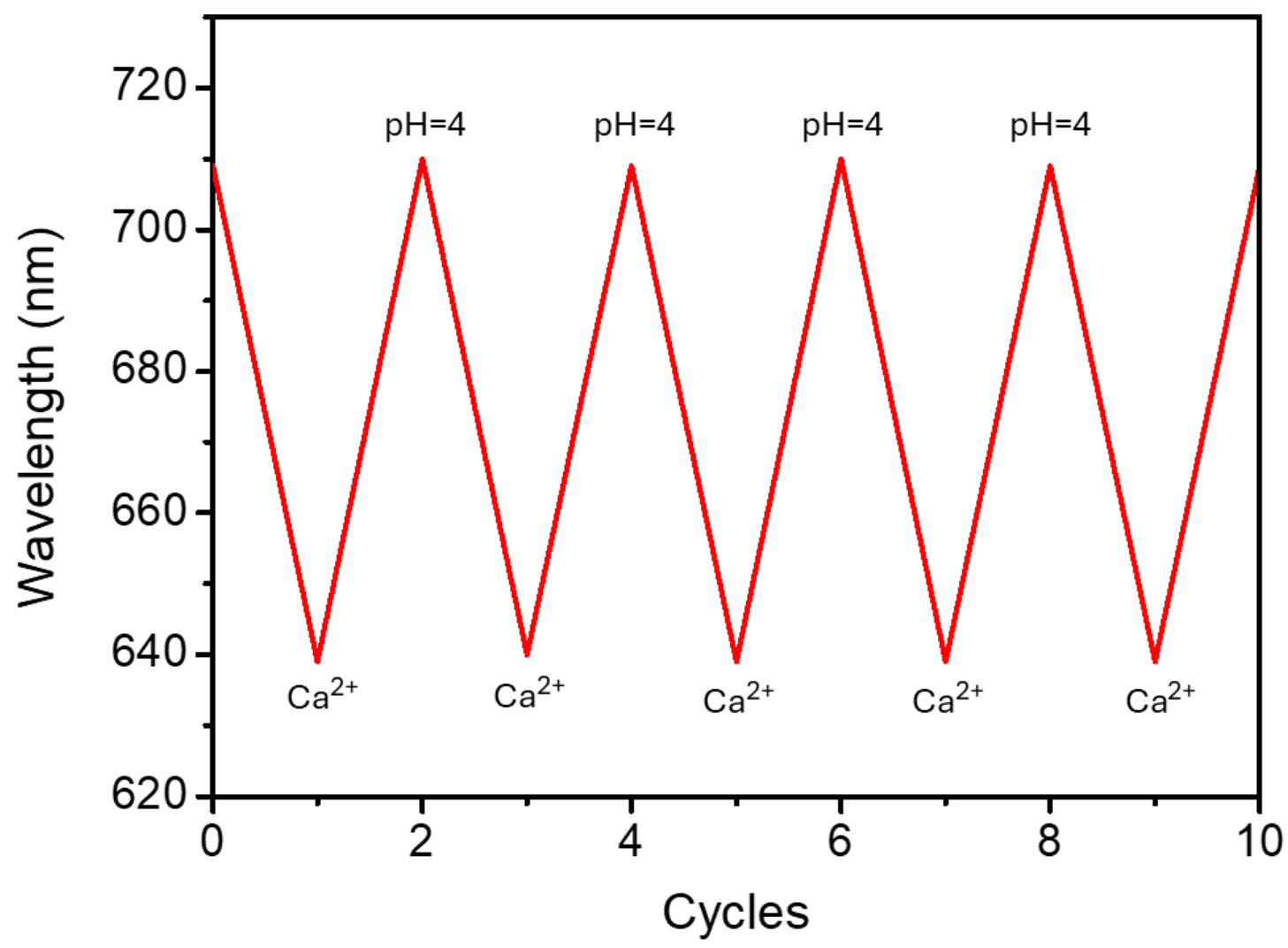

Figure S15. Reflection band evolution during repeated cycles of: Na_2HPO_4/NaH_2PO_4 buffer (pH=8) / 0.10 M Ca^{2+} solution / 1 M NaOAc/AcOH (pH=4). Mixture: E7 (60 wt. %), S811 (20 wt. %), RM257 (10 wt. %), 6OBAM (10 wt. %), BAPO, and BHT.

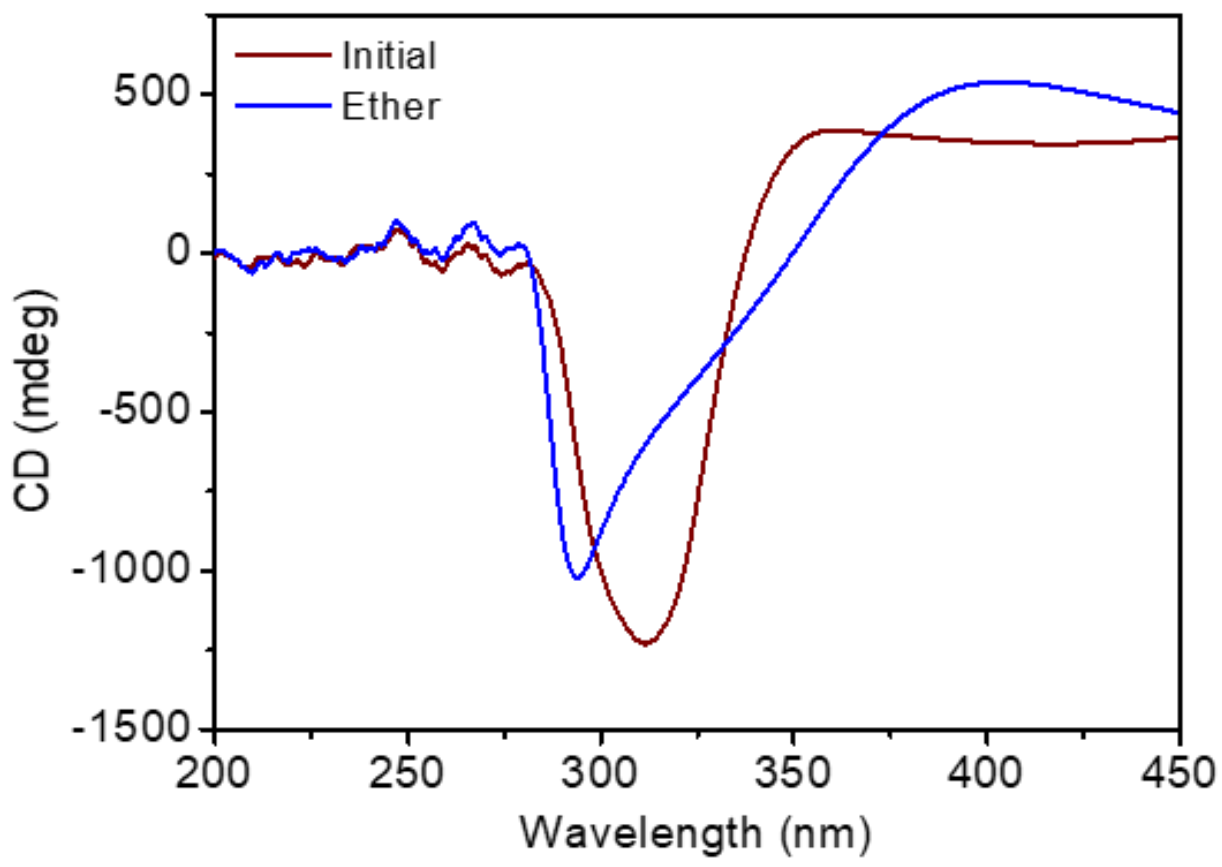

Figure S16. CD spectra of the pristine state (solid red) and after immersion in ether (solid blue). Mixture composition: E7 (60 wt. %), RM257 (20 wt. %), S811 (10 wt. %), 6OBAM (10 wt. %), BAPO, and BHT.

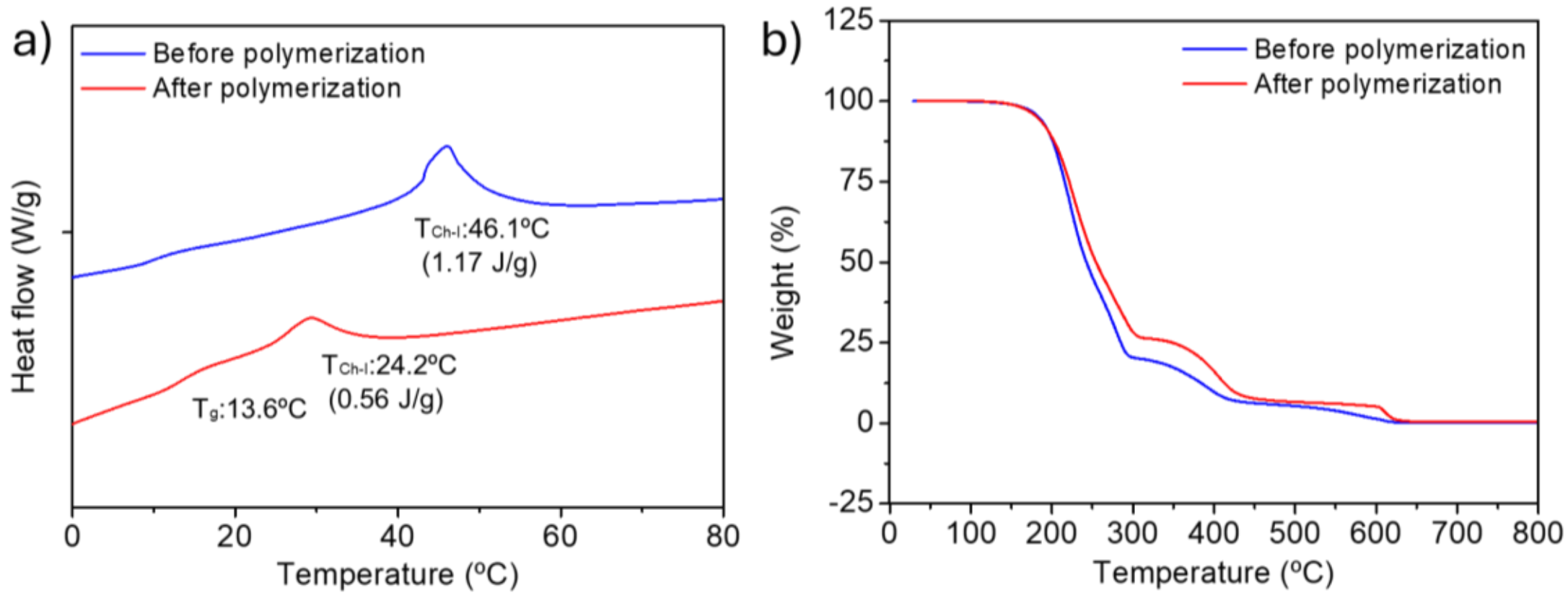

Figure S17. **a)** DSC traces (10ºC/min, exo down) of a LC mixture of E7 (60 wt. %), S811 (20 wt. %), RM257 (15 wt. %), AZO6MA (5 wt. %), BAPO, and BHT before (blue) and after (red) photopolymerization. **b)** TGA traces of the mixture in a) before (blue) and after (red) photopolymerization.

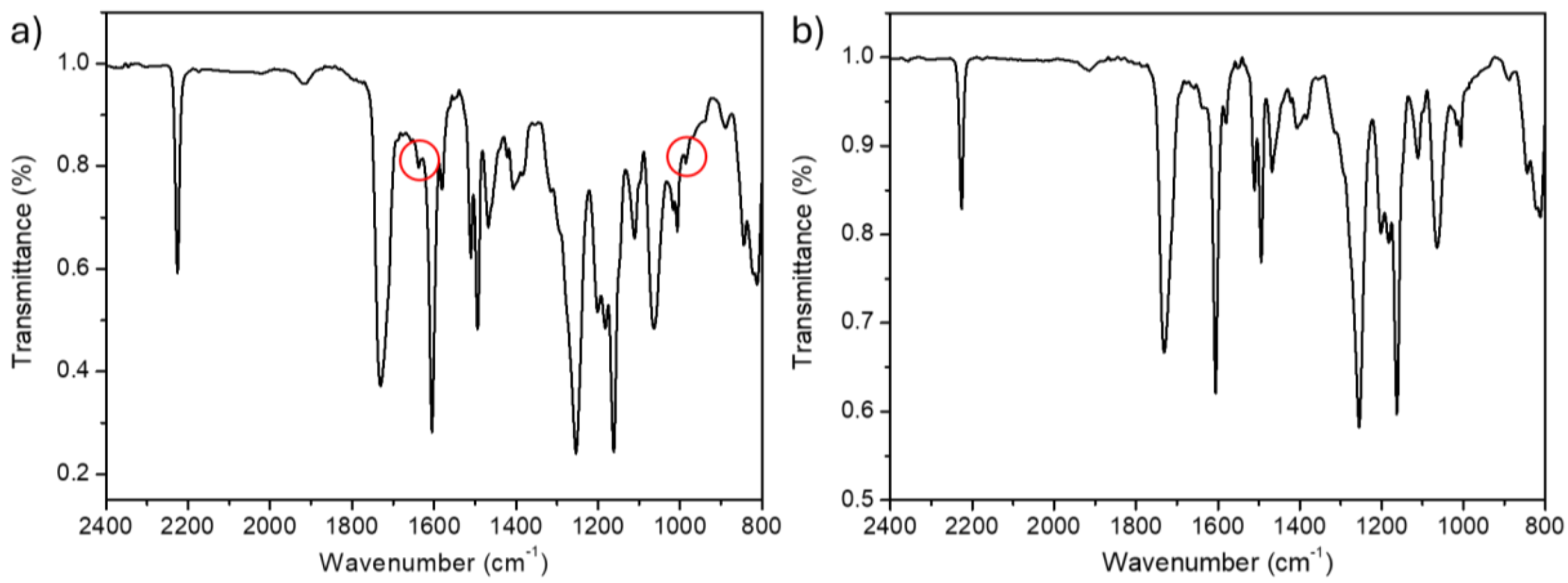

Figure S18. FTIR spectra of: **a)** a LC mixture of E7 (60 wt. %), S811 (20 wt. %), RM257 (15 wt. %), AZO6MA (5 wt. %), BAPO, and BHT before photopolymerization. **b)** the same mixture as in a) after photopolymerization.

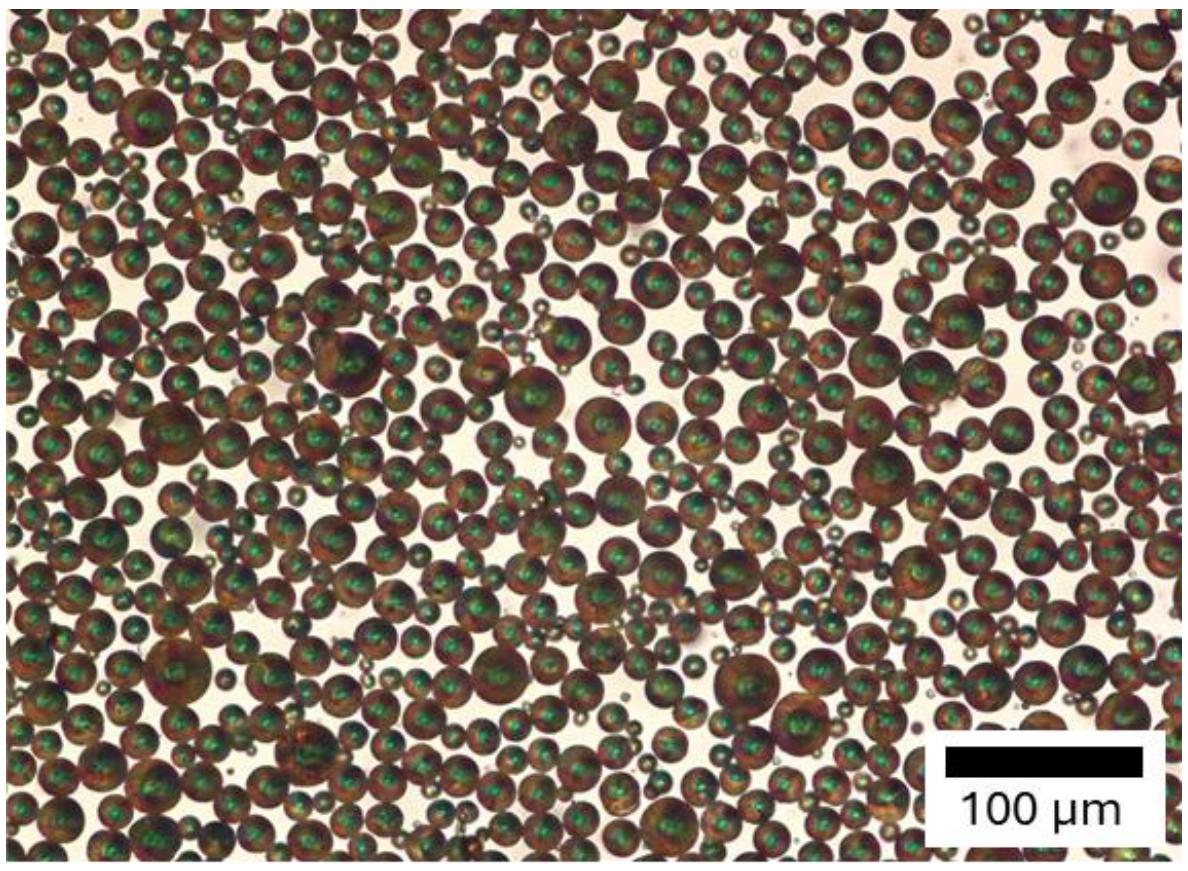

Figure S19. POM images of the UV light-responsive CLC emulsions in a 1 wt. % PVA aqueous solution. LC mixture of E7 (60 wt. %), S811 (20 wt. %), RM257 (15 wt. %), AZO6MA (5 wt. %), BAPO, and BHT.

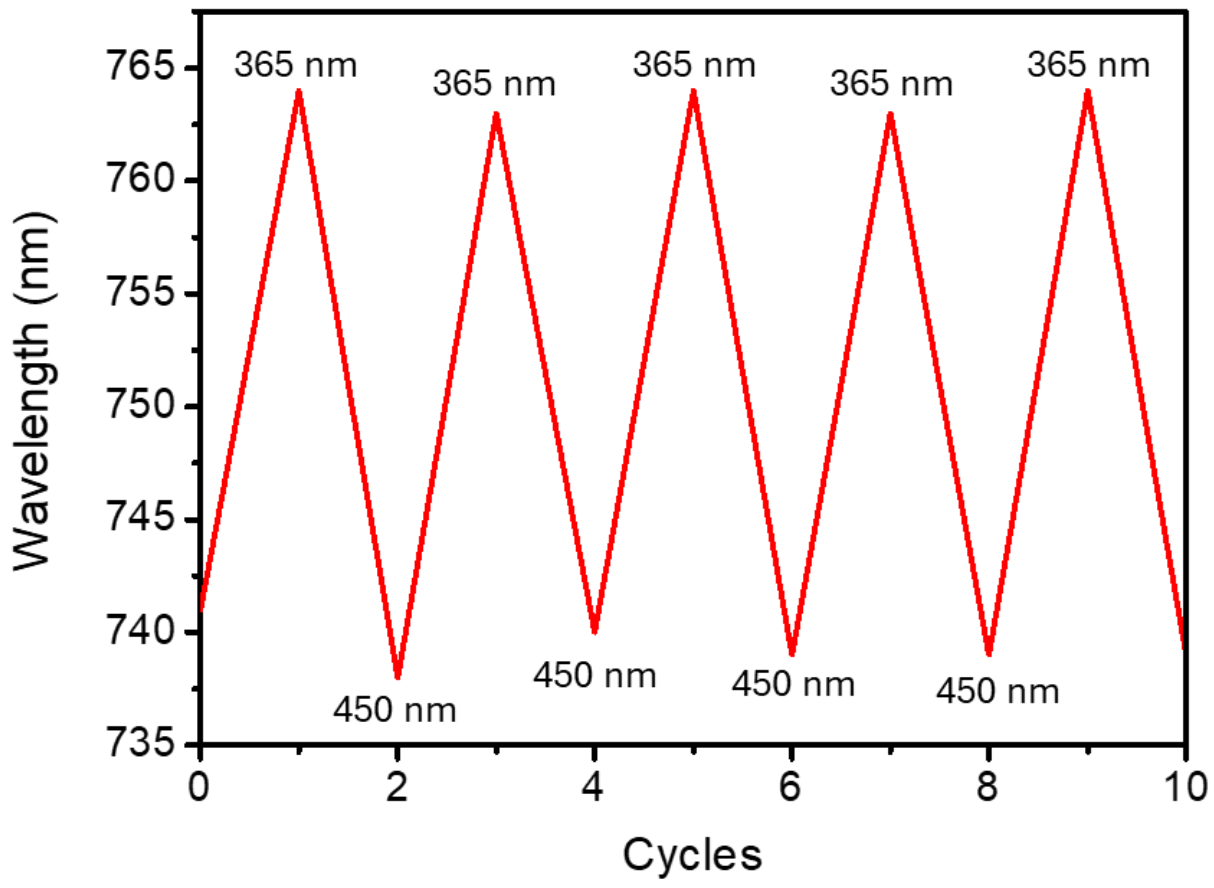

Figure S20. Reflection band evolution during repeated irradiation cycles between 365 - 450 nm. LC mixture of E7 (60 wt. %), S811 (20 wt. %), RM257 (15 wt. %), AZO6MA (5 wt. %), BAPO, and BHT.

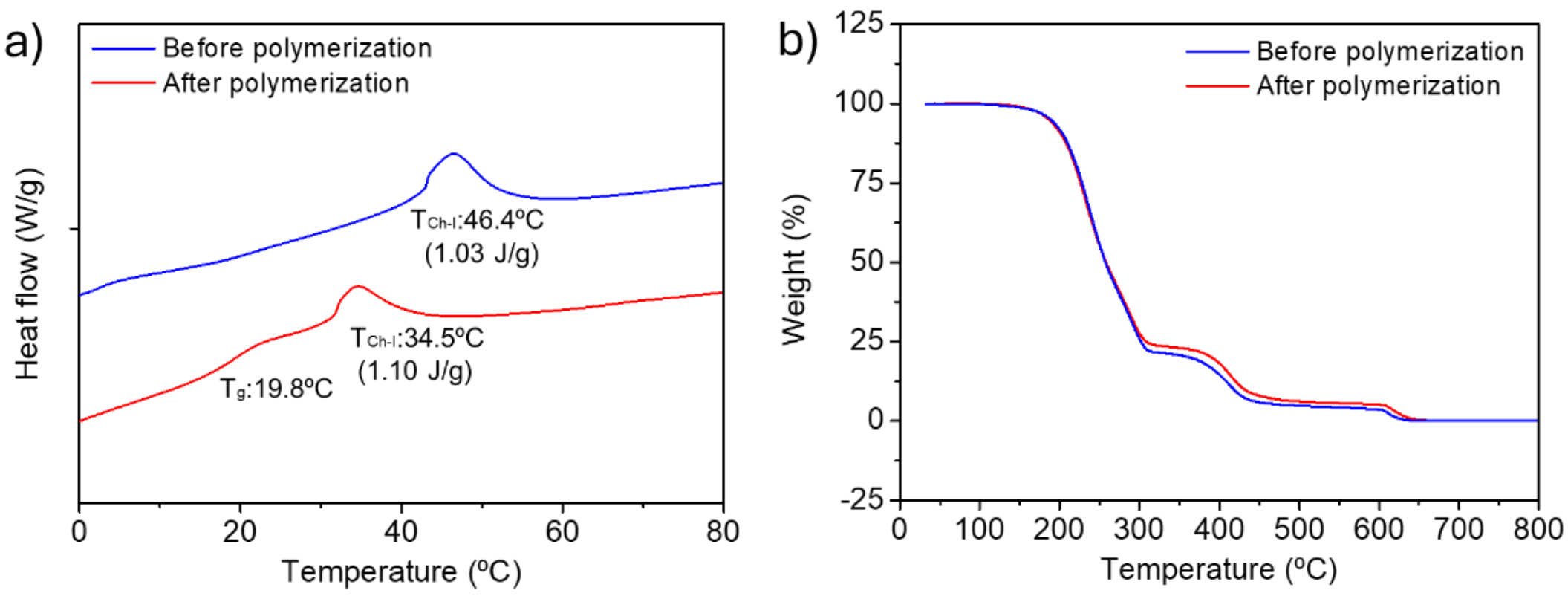

Figure S21. **a)** DSC traces (10°C/min, exo down) of a LC mixture of E7 (60 wt. %), S811 (20 wt. %), RM257 (19.9 wt. %), DR1A (0.1 wt. %), BAPO, and BHT before (blue) and after (red) photopolymerization. **b)** TGA traces of the mixture in a) before (blue) and after (red) photopolymerization.

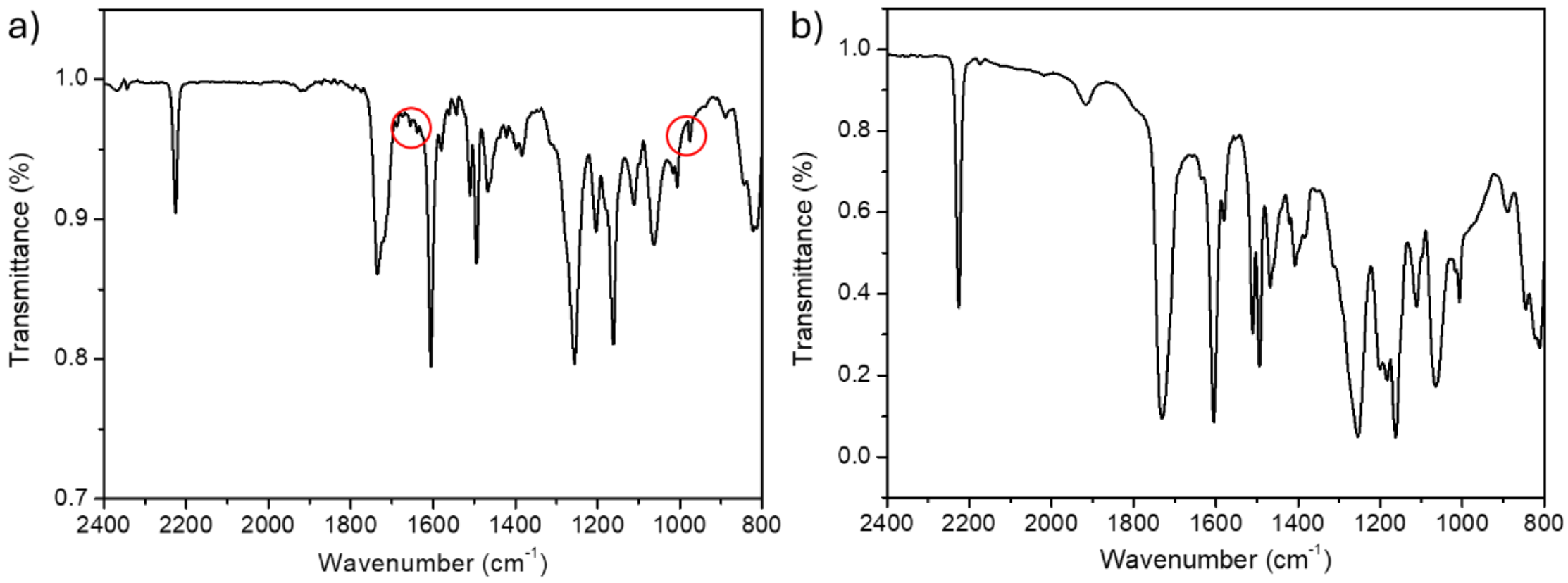

Figure S22. FTIR spectra of: **a)** a LC mixture of E7 (60 wt. %), S811 (20 wt. %), RM257 (19.9 wt. %), DR1A (0.1 wt. %), BAPO, and BHT before photopolymerization. **b)** same mixture as in a) after photopolymerization.

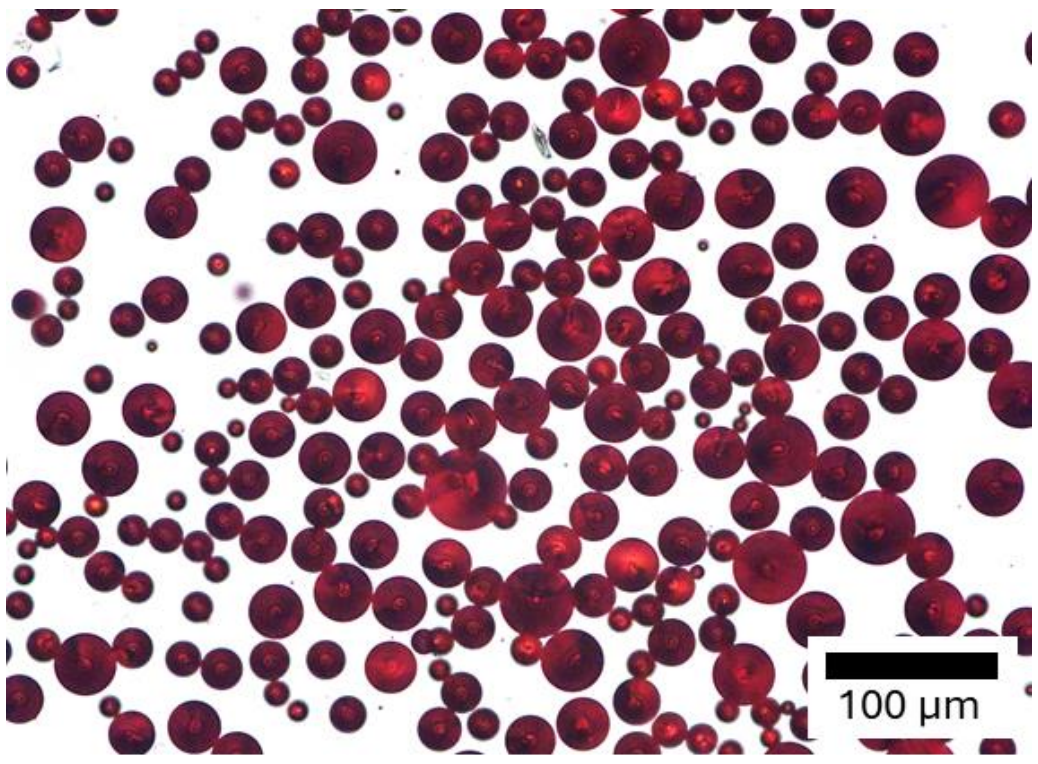

Figure S23. POM images of the UV light-responsive CLC emulsions in a 1 wt. % PVA aqueous solution. LC mixture of E7 (60 wt. %), S811 (20 wt. %), RM257 (19.9 wt. %), DR1A (0.1 wt. %), BAPO, and BHT.

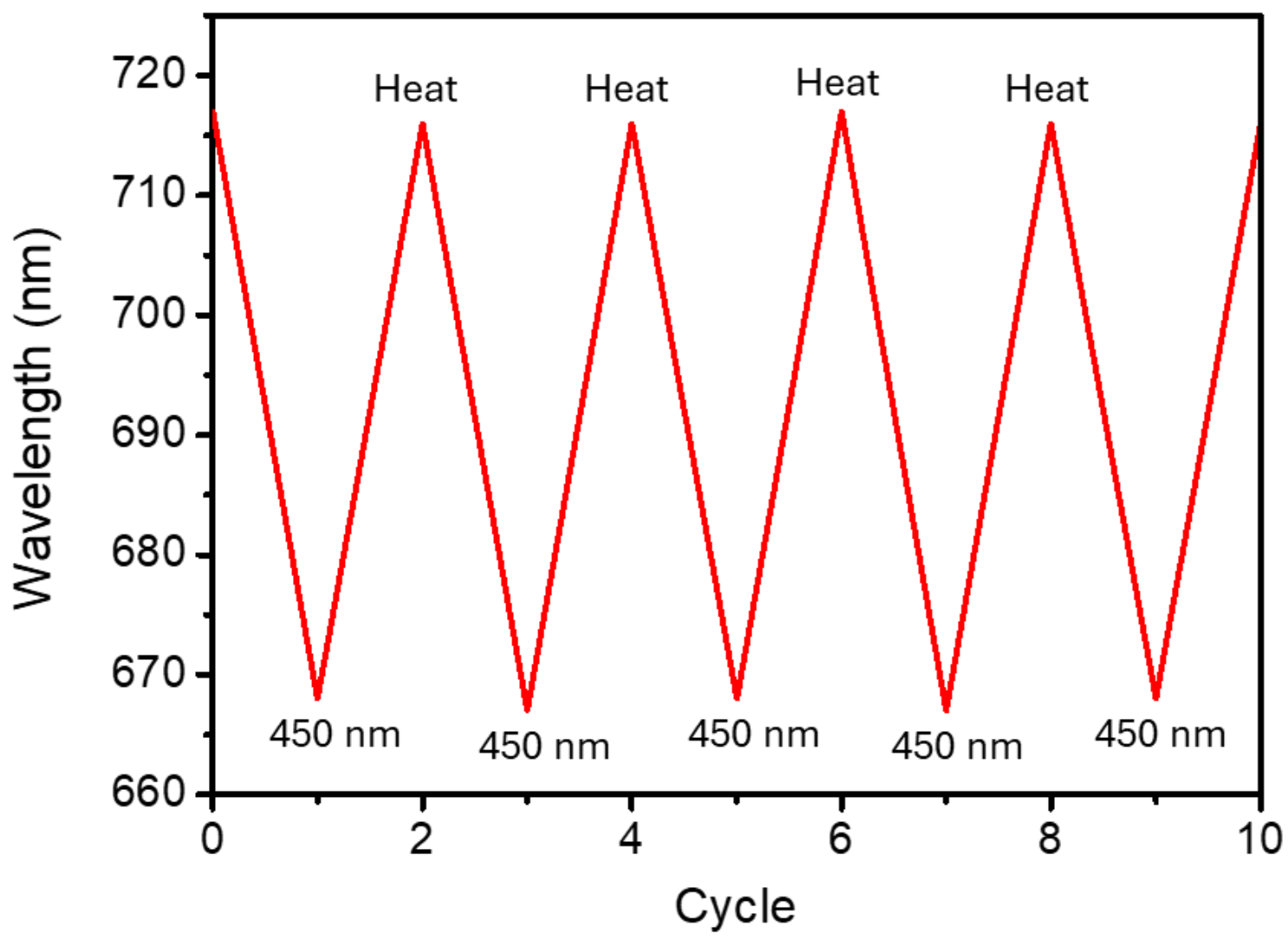

Figure S24. Reflection band evolution during repeated cycles: irradiation 450 nm – mild heating. LC mixture of E7 (60 wt. %), S811 (20 wt. %), RM257 (19.9 wt. %), DR1A (0.1 wt. %), BAPO, and BHT.
